# Macrophage‐Mediated Transport of Insoluble Indirubin Induces Hepatic Injury During Intestinal Inflammation

**DOI:** 10.1002/advs.202502993

**Published:** 2025-06-23

**Authors:** Yiqi Xu, Jingchun Shi, Heung‐Lam Mok, Cheng Lyu, Junbang Chen, Chunhua Huang, Hongyan Qin, Chengyuan Lin, Hor‐Yue Tan, Zhaoxiang Bian

**Affiliations:** ^1^ Centre for Chinese Herbal Medicine Drug Development Hong Kong Baptist University Hong Kong SAR 999077 China; ^2^ School of Chinese Medicine Hong Kong Baptist University Hong Kong SAR 999077 China; ^3^ Department of Pharmacy First Hospital of Lanzhou University Lanzhou 730000 China

**Keywords:** indirubin, liver injury, liver‐gut axis, macrophage extracellular traps, ulcerative colitis

## Abstract

Many plant‐derived bioactive molecules with low solubility and permeability can cause hepatocyte injury. However, the mechanism by which they induce hepatic damage without passive diffusion into the hepatic circulatory system remains unclear. This study demonstrate that indirubin, the main component of indigo naturalis with poor aqueous solubility, predisposes mice with chronic colitis to hepatic injury. This closely mimics the hepatic damage commonly observed in ulcerative colitis patients treated with indigo naturalis. Upon administration, indirubin is detected in the plasma, Peyer's patches, and hepatic tissue, with its distribution linked to macrophage infiltration into the liver. Ablation of macrophages significantly reduces indirubin accumulation and attenuates elevated hepatic transaminases in mice with chronic colitis. Mechanistically, macrophages internalize and transport indirubin aggregates from Peyer's patches through the circulatory system to the livers. This internalization activates the NLRP3 inflammasome, leading to the formation of macrophage extracellular traps (METs), which contribute to oxidative stress‐induced liver injury. The study identifies indirubin as a potentially toxic component of indigo naturalis that provokes METs‐mediated oxidative damage. Additionally, the findings reveal a novel transport pathway for poorly soluble molecules to reach the liver via uptake by macrophages within Peyer's patches.

## Graphical abstract

Plant‐derived bioactive molecules with low solubility and permeability induce hepatocyte injury, though the mechanisms driving their hepatic effects remain poorly understood. This study identifies a novel transport pathway in which poorly soluble indirubin accumulates in the liver via macrophage‐mediated uptake in Peyer's patches, exacerbating hepatic injury in chronic colitis mice. This process mirrors clinical observations in ulcerative colitis patients using such herb.

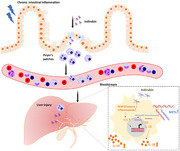

## 1 Introduction

Drug‐induced liver injury (DILI) is a well‐documented adverse effect associated with the use of certain pharmaceutical drugs and herbal supplements. While the incidence of such injury is relatively low, it accounts for ≈50% of acute liver failure cases.^[^ ^1^ ^]^ DILI may arise from the direct effects of the drug or its metabolites, or indirectly via immune‐mediated mechanisms.^[^ ^2^ ^]^ One key determinant of a drug's potential to cause toxic effects or unintended interactions in the liver is its oral absorption. Highly soluble and permeable drugs readily dissolve in gastrointestinal fluid, permeate the intestinal wall, and enter the bloodstream, where they are ultimately absorbed by hepatocytes.^[^ ^3^ ^]^ Thus, there is a positive correlation between a drug's solubility and permeability with the latency and severity of hepatocellular injury.^[^ ^4^ ^]^ Nevertheless, our current understanding of the propensity of poorly soluble drugs to induce liver damage remains limited. Drug solubility is a particular concern in traditional herbal medicine. Unlike synthetic drugs, many plant‐derived bioactive molecules are poorly water‐soluble, exhibit low oral bioavailability, or both. An enduring puzzle in herbal medicine research is how such compounds exert systemic effects despite these limitations. This has led us and others to propose alternative absorption pathways, such as transcytosis through gut M cells into Peyer's patches.^[^ ^5^ ^]^ However, it remains unclear how insoluble drugs pass beyond Peyer's patches to enter systemic circulation.

Our study focuses on indigo naturalis (IN), a dried powder primarily derived from the stems or leaves of *Baphicacanthus cusia* (Nees) Bremek.,^[^ ^6^ ^]^ recognized for its therapeutic efficacy in treating ulcerative colitis (UC).^[^ ^7^, ^8^ ^]^ The principal bioactive ingredients in IN are indigo and indirubin, both nearly insoluble in water and exhibiting low oral bioavailability. Short‐term IN administration demonstrated a promising clinical response rate versus placebo (69.6% vs 13.6%), yet severe therapy‐related adverse events – particularly hepatic injury – have been reported in patients. This complication typically manifests within the first two months of treatment, often leading to therapy discontinuation.^[^ ^9^ ^]^ A recent study showed that patients discontinuing IN therapy experienced higher UC relapse rates within 52 weeks.^[^ ^10^ ^]^ Indigo and indirubin exhibit high‐affinity binding to the aryl hydrocarbon receptor (AHR), a validated target in UC due to its dual role in epithelial homeostasis^[^ ^11^ ^]^ and promotion of regulatory T cells.^[^ ^12^ ^]^ Paradoxically, AHR agonism also induces hyperacute toxicity in preclinical models.^[^ ^13^ ^]^ Despite evident mechanistic links between the IN's therapeutic effects and adverse events, the hepatotoxic potential of its insoluble constituents remains mechanistically unexplained, hindering clinical utility and risk mitigation.

In this study, we demonstrate that indirubin is the primary component in IN responsible for predisposing mice with chronic colitis to hepatic injury. We show that indirubin is predominantly transported to the liver via macrophages located in Peyer's patches, and macrophage depletion ameliorates this indirubin‐induced hepatic injury. Physiologically, this represents a new route for the delivery of water‐insoluble drugs. Mechanistically, hepatic indirubin accumulation triggers macrophage infiltration, induces the formation of macrophage extracellular traps (METs), and subsequently drives METosis – a specific form of cell death – leading to hepatic oxidative damage. Our findings underscore the pivotal role of macrophages in mediating the transport of insoluble molecules during chronic intestinal inflammation. A deeper understanding of how insoluble molecules are directed to hepatocytes under inflammatory conditions could inform the development of safer therapeutics with reduced hepatotoxic risk.

## 2 Results

### 2.1 Long‐Term Administration of a Sub‐Lethal Dose of Indirubin Predisposes Mice With Colitis to Hepatic Injury

**Figure 1:**
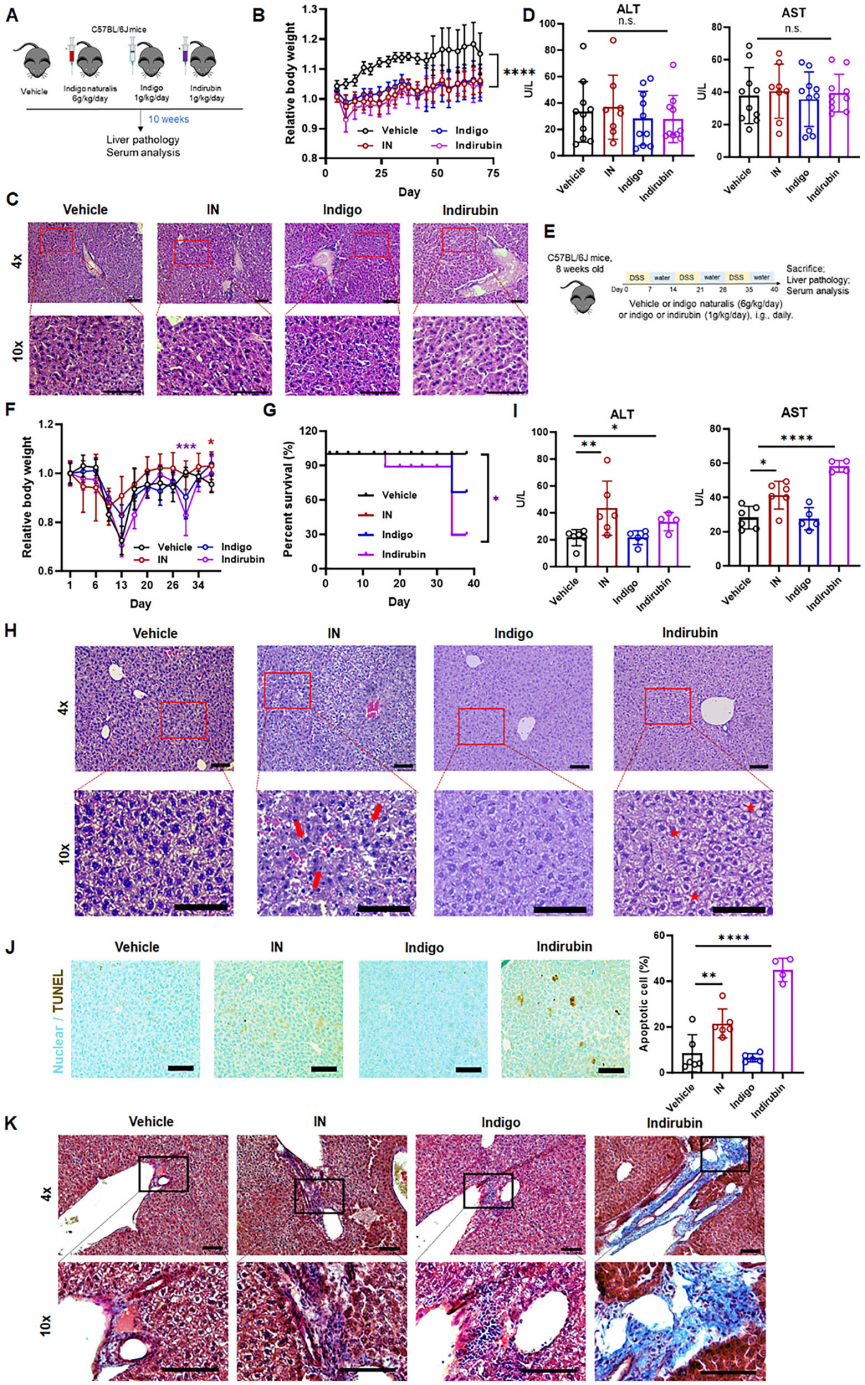
Long‐term administration of a sub‐lethal dose of indirubin predisposes colitis mice to hepatic injury. A) Schematic illustration of animal experiments with indigo naturalis (IN) and its compounds. C57BL/6J mice were intragastrically administrated with indigo (1 g kg^−1^), indirubin (1 g kg^−1^), or IN (6 g kg^−1^) for 10 weeks. B) Relative body weight change; C) H&E staining of liver tissues from mice treated with IN, indigo, or indirubin. Scale bar: 100 µm. D) Serum ALT and AST levels. Data are expressed as mean ± SD (*n * = 10 mice/group). E) Schematic illustration of IN and compounds administration in the DSS‐induced chronic colitis model. Mice received three cycles of 1.8% DSS in drinking water. F) Relative body weight change; (G) Kaplan‐Meier survival curve during experimental period and H) H&E staining of liver sections from colitis mice treated with IN, indigo, or indirubin. Scale bar: 100 µm. I) Serum ALT and AST levels. Data are expressed as mean ± SD (n  =  6 mice/group). J) TUNEL staining of liver sections; and K) Masson's trichrome staining showing collagen deposition in liver sections from mice. Scale bar: 100 µm. n. s., not significant; *p < 0.05; **p < 0.01; ****p < 0.0001 (Multiple *t‐*test or one‐way ANOVA).

Hepatic injury is a common adverse event in UC patients who receive IN for two months or longer.^[^ ^7^, ^14^ ^]^ To systematically evaluate potential IN‐induced hepatotoxicity, we administered IN (6 g kg^−1^, ≈10 times the human equivalent dose ^[^ ^8^ ^]^) or its major constituents – indigo and indirubin (1 g kg^−1^ each, ≈30 times the human equivalent dose ^[^ ^15^ ^]^) – to healthy C57BL/6J mice for ten weeks (**Figure** **1**A). Compared to vehicle controls, intervention groups exhibited significantly slower body weight gain (Figure 1B). However, hepatic tissues showed no histological abnormalities, and serum alanine aminotransferase (ALT) and aspartate aminotransferase (AST) levels remained unaltered post‐intervention (Figure 1C,D; Figure S1A–C, Supporting Information). These findings indicate minimal hepatotoxicity of IN and its primary components in healthy mice.

Given the reported association between IN administration and hepatic abnormalities UC patients, we extended our investigation to a chronic colitis mouse model established through repeated dextran sulfate sodium (DSS) challenges.^[^ ^16^ ^]^ Mice received high dose IN, indigo, or indirubin for six weeks (Figure 1E). Indirubin treatment significantly reduced body weight by day 30, coinciding with the exacerbation of DSS‐induced disease (Figure 1F). Additionally, three mice treated with indirubin required early euthanasia on days 16 and 34 due to severe illness (Figure 1G). Hepatic histology revealed abnormalities characterized by heterogeneous foci of cellular alterations, including clear cells and inflammatory infiltrates (Figure 1H). Mice that received IN and indirubin exhibited significantly elevated ALT and AST levels versus vehicle controls (Figure 1I). Consistent with this, TUNEL^+^ apoptotic cells and trichrome‐stained collagen deposition increased in the livers of these mice (Figure 1J,K). These findings indicate that indirubin, a component of IN, predisposes mice with chronic colitis to an increased risk of hepatic injury.

### 2.2 Accumulation of Indirubin in the Peyer's Patches and Hepatic Tissue of Mice with Chronic Colitis

**Figure 2:**
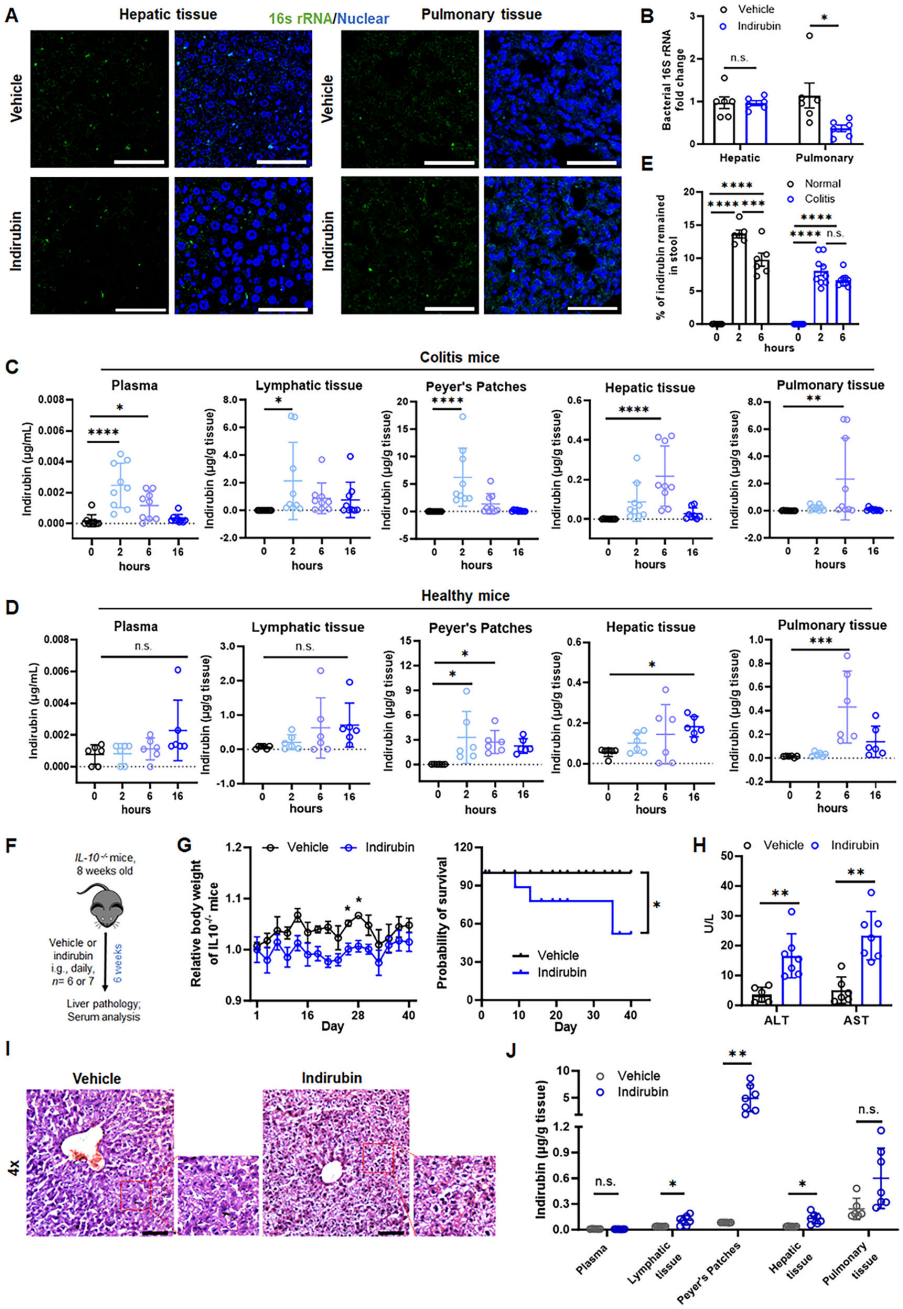
Accumulation of indirubin in the Peyer's patches and hepatic tissue of mice with chronic colitis. A) Representative fluorescence images of 16S rRNA (green) in hepatic and pulmonary tissues from colitis mice. Nuclei are counterstained with DAPI (blue). Scale bars  =  50 µm. B) Fold changes in 16S rRNA gene levels in hepatic and pulmonary tissues from colitis mice treated with vehicle or indirubin. Data are expressed as mean ± SEM (*n * = 6 mice/group). Pharmacokinetic profile of indirubin in plasma and tissues at indicated time points (0, 2, 6, and 16 h) in C) colitis and D) healthy mice. (colitis, *n * = 9 mice/group; healthy, *n * = 6 mice/group). E) Fecal excretion of indirubin quantified by LC‐MS/MS. Data are expressed as mean ± SEM (colitis, *n * = 9 mice/group; healthy, *n * = 6 mice/group). F) Experimental scheme for IL‐10‐knockout spontaneous colitis model. G) Body weight changes (left) and survival analysis (right); H) serum ALT and AST levels and I) representative H&E‐stained liver sections from IL‐10‐knockout mice treated with vehicle or indirubin. Scale bar: 100 µm. J) Tissue distribution of indirubin in IL‐10‐knockout mice. Data are expressed as mean ± SD (Vehicle, n  =  6 mice/group; Indirubin, *n * = 7 mice/group). n.s., not significant; *p < 0.05; **p < 0.01; ***p < 0.001; ****p < 0.0001 (Student's *t‐*test or one‐way ANOVA).

A disrupted gut‐vascular barrier in colitis allows systemic bacteria dissemination to the liver, causing localized injury.^[^ ^17^ ^]^ To examine whether colonic inflammation drives bacterial DNA accumulation in the liver, we quantified 16s rRNA in hepatic and pulmonary tissues of chronic colitis mice. Despite increased vascular permeability (Figure S2A, Supporting Information), bacterial DNA was barely detected in these tissues (**Figure** **2**A). Levels of hepatic and pulmonary bacterial DNA and lipopolysaccharides (LPS) in indirubin‐treated colitis mice remained comparable to vehicle controls (Figure 2B; Figure S2B, Supporting Information). Hepatic expression of microbial metabolite receptors, including toll like receptors (TLRs) and G protein‐coupled receptors (GPCRs), showed no significant increase with indirubin treatment (Figure S2C, Supporting Information). Moreover, indirubin‐treated pseudo‐germ‐free (PGF) mice exhibited higher mortality than vehicle‐treated counterparts, suggesting gut bacteria may protect against indirubin‐induced injury (Figure S2D, Supporting Information).

Since liver injury appeared independent of bacterial accumulation, we next addressed whether indirubin could circulate through the bloodstream and reach metabolic tissues. After oral administration in colitis mice, indirubin was detectable at 2 h in plasma (0.002 µg mL^−1^) and colonic lymphatic tissue (2.13 µg g^−1^), with the highest concentration found in Peyer's patches (6.22 µg g^−1^). Hepatic (0.22 µg g^−1^) and pulmonary (2.33 µg g^−1^) concentrations peaked at 6 h (Figure 2C). In healthy mice, Peyer's patches showed maximal indirubin at 2 h postadministration, but levels were negligible in the plasma or lymphatic tissues. Hepatic and pulmonary concentrations were significantly lower or peaked later than in colitis mice (Figure 2D). Additionally, fecal excretion at 2 h was 15% in healthy mice, in contrast to less than 10% in colitis mice (Figure 2E). Due to the lack of modifiable functional groups for covalent binding to indirubin, we further examined the systemic distribution of FITC‐labelled synthetic particles (5–7.9 µm; size‐matched to indirubin) post‐administration. The fluorescence signal was detected in colonic lymphatic tissue, Peyer's patches, lungs, and liver (Figure S2E,F, Supporting Information). Notably, Peyer's patches exhibited the earliest and highest fluorescence intensity, consistent with indirubin's distribution pattern, suggesting particle accumulation precedes hepatic migration.

To determine whether the differential tissue distribution of indirubin extends beyond DSS‐induced colitis, we evaluated its effect in IL‐10 knockout mice, which spontaneously develop colitis by 6–12 weeks of age (Figure 2F). Six‐week daily administration of a sublethal indirubin dose to IL‐10 knockout mice promoted weight loss and overall health decline, prompting the need for early euthanasia (Figure 2G). Notably, indirubin‐treated mice exhibited significant hepatic damage, evidenced by elevated levels of ALT and AST, and histological abnormalities (Figure 2H,I). This phenotype mirrored observations in DSS‐induced chronic colitis mice, where indirubin accumulated robustly in both the Peyer's patches and hepatic tissue (Figure 2J). Collectively, these results demonstrate preferential accumulation of indirubin in the livers and lungs of mice with colitis compared to healthy mice. The transport kinetics indicate sequential deposition, whereby initial uptake occurs in Peyer's patches, followed by systemic dissemination to the liver and lungs via circulation.

### 2.3 A Critical Role of Peyer's Patches Macrophage in Processing Indirubin

**Figure 3:**
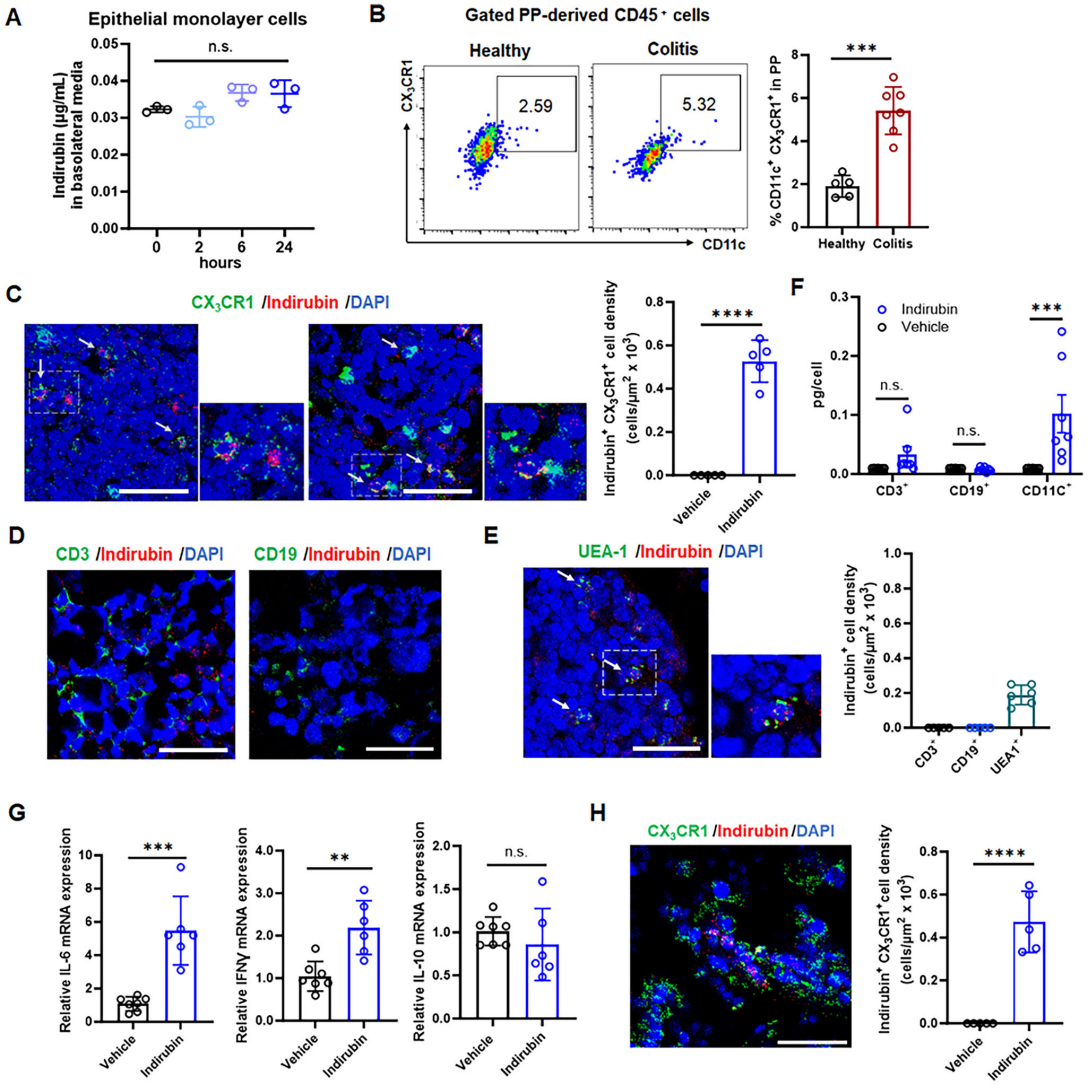
The critical role of Peyer's patch macrophages in indirubin processing. A) Indirubin concentration in basolateral media after co‐culture with epithelial monolayers over time. Data are expressed as mean ± SD (*n * = 3). B) Flow cytometry analysis of CD11c^+^ CX_3_CR1^+^ macrophages in Peyer's patches of healthy and colitis mice. Data are expressed as mean ± SD (colitis, *n * = 7 mice/group; healthy, *n * = 5 mice/group). Immunofluorescence staining of C) CX_3_CR1^+^ macrophages, D) CD3^+^ T and CD19^+^ B cells, E) UEA‐1^+^ M cells in Peyer's patches of indirubin‐treated chronic colitis mice. Nuclei were counterstained with DAPI. Scale bar = 50 µm. Quantiification of indirubin colocalization (right panel). F) Internalized indirubin in CD3^+^ T, CD19^+^ B cells and CD11c^+^ macrophages from Peyer's patches. Data are expressed as mean ± SD (*n * = 7). G) The mRNA expression of pro‐inflammatory genes *IL‐6* and *IFNγ* and anti‐inflammatory *IL‐10* in Peyer's patches. Data are expressed as mean ± SD (vehicle, *n * = 7; indirubin, *n * = 6). H) Immunofluorescence staining of CX_3_CR1^+^ macrophages colocalized with indirubin in Peyer's patches of IL‐10 knockout mice. Quantification of colocalization (right panel). n. s., not significant; *p < 0.05; **p < 0.01; ***p < 0.001; ****p < 0.0001 (Student's *t‐*test or one‐way ANOVA).

Indirubin is water insoluble,^[^ ^18^ ^]^ which prevents its ability to diffuse passively across the intestinal cell monolayer (**Figure** **3**A). We hypothesize that indirubin aggregates undergo transcytosis through M cells in Peyer's patches,^[^ ^5^ ^]^ followed by systemic transport by migrating immune cells. To identify relevant immune populations in colitis mice, we profiled the immune cells in the Peyer's patches potentially harboring indirubin. Within the subepithelial dome, populated by mononuclear phagocytes (MNPs) including dendritic cells and macrophages,^[^ ^19^ ^]^ we observed a marked increase in CD11c^+^ CX_3_CR1^+^ macrophages during colitis compared to healthy mice (Figure 3B). Notably, CX_3_CR1^+^ macrophages rapidly internalized indirubin within 2 h of administration (Figure 3C), unlike other immune cells (Figure 3D). As anticipated, UEA‐1^+^ M cells also showed indirubin intake (Figure 3E). Microscopy revealed macrophages contained insoluble indirubin aggregates (predominantly low µm range), though smaller particles below detection limits may exist. LC‐MS/MS quantification of FACs‐sorted cells confirmed significantly higher indirubin levels in CD11C^+^ macrophages (0.1021 pg cell^−1^) than in CD3^+^ T and CD19^+^ B cells (Figure 3F).

Unlike intestinal phagocytes, Peyer's patches macrophages retain pro‐inflammatory functions, primarily defending against foreign particles and initiating adaptive immunity, rather than acquiring anti‐inflammatory properties.^[^ ^20^ ^]^ Consistent with this, indirubin‐treated colitis mice exhibited upregulated pro‐inflammatory IL‐6 and IFN‐γ expression in Peyer's patches compared to vehicle controls, while anti‐inflammatory IL‐10 expression remained unchanged (Figure 3G). Extending these observations to IL‐10 knock‐out mice, we detected pronounced CX_3_CR1^+^ macrophage infiltration in Peyer's patches, with significant indirubin colocalization (Figure 3H). This indicates that IL‐10 deficiency does not impair macrophage‐mediated indirubin uptake and transport. Taken together, these findings support a mechanism whereby poorly soluble indirubin is transported to the liver following uptake by macrophages in Peyer's patches.

### 2.4 Indirubin‐induced Hepatic Injury in Colitis Requires the Presence of Macrophages from Peyer's Patches

**Figure 4:**
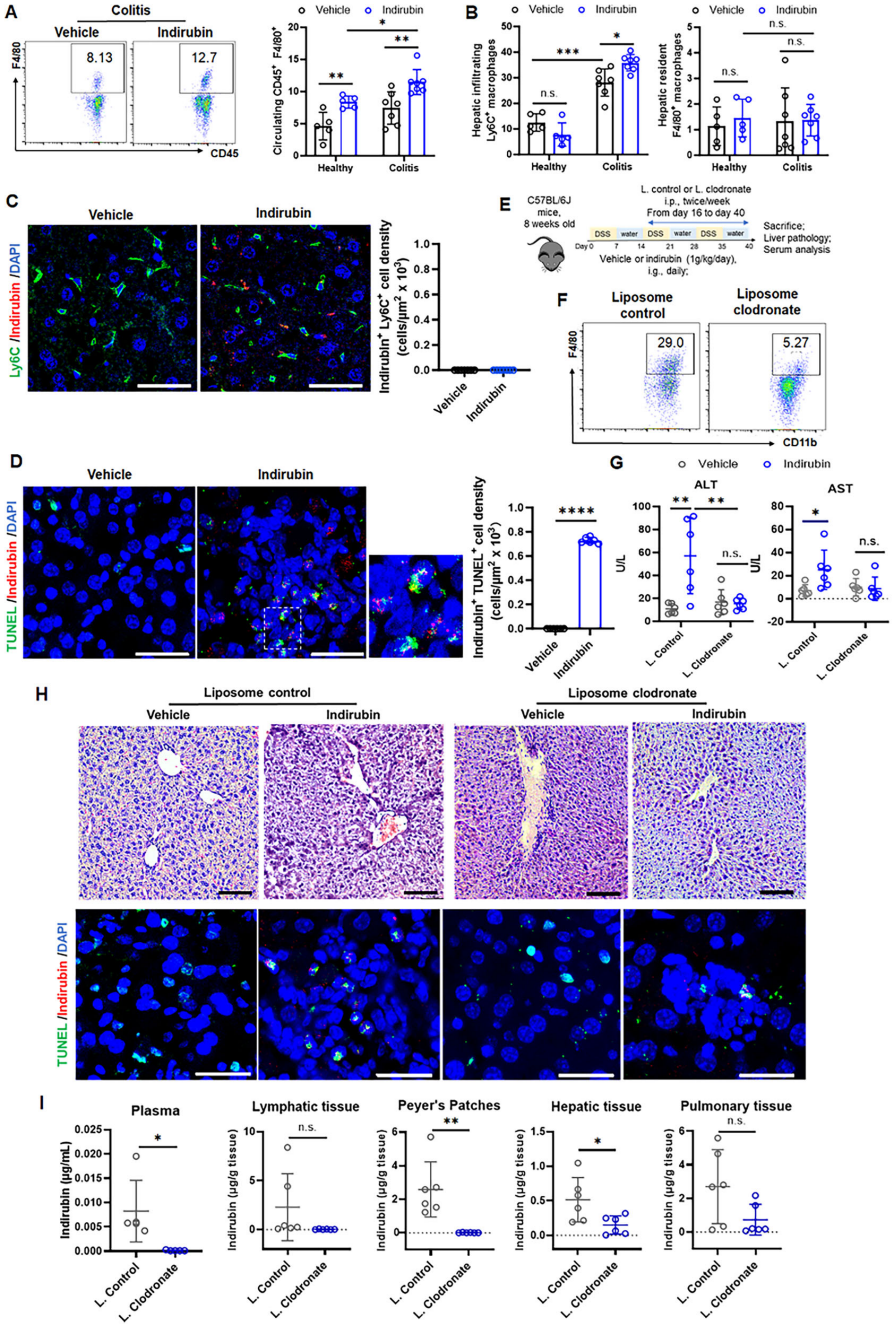
Phagocytosis of indirubin crystals promotes macrophage activation. Flow cytometry analysis of A) circulating CD45^+^ F4/80^+^ macrophages; B) hepatic infiltrating Ly6C^+^ macrophages and resident F4/80^+^ macrophages in healthy and colitis mice with vehicle or indirubin treatment. Data are expressed as mean ± SD (colitis, *n * = 7 mice/group; healthy, *n * = 5 mice/group). Immunofluorescence staining of C) Ly6C^+^ macrophages and D) TUNEL^+^ apoptotic cells in liver tissues. Scale bar: 50 µm. E) Schematic illustration of macrophage depletion in indirubin‐treated colitis mice. F) Flow cytometry analysis of circulating CD11b^+^ F4/80^+^ macrophages in mice treated with control‐ or clodronate‐loaded liposomes. G) Serum ALT and AST levels in colitis mice post‐macrophage depletion. Data are expressed as mean ± SD (*n * =  6 mice/group). H) H&E staining (top, scale bar: 100 µm) and TUNEL^+^ cells (bottom, scale bar: 50 µm) in liver tissues ± indirubin and macrophage depletion. I) Tissue distribution of indirubin in mice treated with control or clodronate‐loaded liposomes. Data are expressed as mean ± SD (*n * = 6 mice/group). n.s., not significant; *p < 0.05; **p < 0.01; ***p < 0.001; ****p < 0.0001 (Student's *t* test or one‐way ANOVA).

Macrophages are professional phagocytes specialized in recognizing, internalizing, and clearing cellular debris and foreign particles.^[^ ^21^ ^]^ This further prompted us to investigate whether macrophages transport insoluble indirubin aggregates to the liver via circulation. Immune profiling revealed a significant increase in circulating CD45^+^ F4/80^+^ macrophages in both chronic colitis and healthy mice following indirubin administration (**Figure** **4**A). However, only colitis mice developed significant hepatic infiltration of Ly6C^+^ macrophages without changes in resident F4/80^+^ populations (Figure 4B). Notably, while microscopy showed no direct colocalization of indirubin with hepatic macrophages (Figure 4C; Figure S3A, Supporting Information), aggregates localized near TUNEL^+^ apoptotic cells (Figure 4D), suggesting macrophage‐transported indirubin interacts with dying hepatocytes.^[^ ^22^ ^]^

To determine the role of macrophages in indirubin‐induced hepatic injury, we depleted the macrophages using clodronate‐loaded liposomes in colitis mice postindirubin exposure (Figure 4E). This approach successfully reduced the macrophage population by over 90% within 24 h postadministration (Figure 4F). Macrophage ablation in the colitis mice led to decreased levels of ALT and AST, compared to those treated with control liposomes after indirubin administration (Figure 4G). Histological examination revealed no damage, and hepatic TUNEL^+^ apoptotic cells were significantly reduced after macrophage depletion, confirming the essential role of macrophages in indirubin‐induced hepatic injury (Figure 4H). Consistently, macrophage ablation abolished indirubin accumulation in Peyer's patches, liver, and lungs at 2 h post‐administration compared to control liposomes‐treated mice (Figure 4I). Collectively, our data establish macrophages as indispensable transporters that accumulate indirubin aggregates in Peyer's patches and mediate systemic dissemination to the liver and lungs.

### 2.5 Phagocytosis of Indirubin Crystals Triggers Macrophage Inflammatory Responses

**Figure 5:**
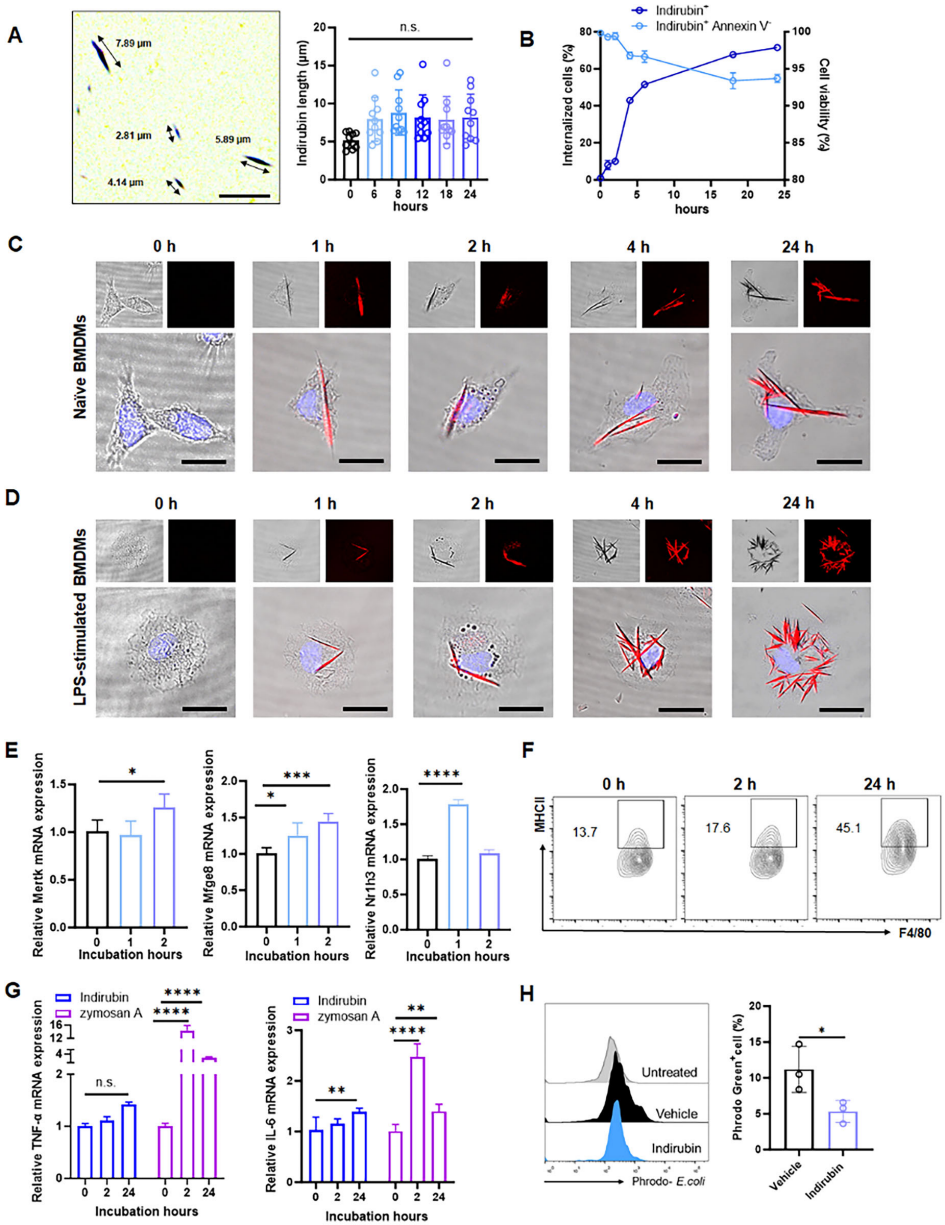
Modest macrophage activation following phagocytosis of indirubin crystals. A) Needle‐like indirubin crystals (left; scale bar: 10 µm) and quantification (right). Data are expressed as mean ± SD (*n * =  10). B) Internalization of indirubin by Annexin V^+^ BMDMs over time, analyzed by flow cytometry. Data are expressed as mean ± SD (*n * =  4). Indirubin uptake by (C) naïve or D) LPS‐induced BMDMs over time. Scale bar: 10 µm. E) Phagocytosis‐related mRNA expression in BMDMs after indirubin treatment. Data are expressed as mean ± SD (*n * =  6). F) The expression of MHCII in F4/80^+^ BMDMs post‐indirubin treatment. G) Inflammatory gene mRNA expression in BMDMs treated with indirubin or zymosan A. Data are expressed as mean ± SD (*n * =  6). H) Quantification of F4/80^+^ phrodo green^+^ BMDMs. Data are expressed as mean ± SD (*n * = 3). n.s., not significant; *p < 0.05; **p < 0.01; ***p < 0.001; ****p < 0.0001 (Student's *t* test or one‐way ANOVA).

Given the observed uptake of indirubin by macrophages in Peyer's patches, we investigated whether its internalization modulates macrophage functionality. Phagocytic capacity, a critical macrophage function, is influenced by particle size and morphology.^[^ ^23^, ^24^ ^]^ Indirubin formed needle‐like crystals (3–8 µm) that aggregated progressively in culture (**Figure** **5**A). Bone marrow‐derived macrophages (BMDMs) internalized 15% of indirubin within 2 h while maintaining viability above 95%. By 24 h, uptake efficiency increased to 70%, with viability dropping marginally to above 90% (Figure 5B). Further microscopy confirmed a time‐dependent accumulation of intracellular indirubin (Figure 5C). Furthermore, LPS‐induced pro‐inflammatory BMDMs exhibited significantly higher indirubin internalization efficiency compared to naïve BMDMs (Figure 5D), consistent with the observations that activated macrophages display augmented phagocytic capacity.^[^ ^25^ ^]^ This effect was cell type‐specific, as no similar uptake was observed in other cell types (Figure S3B,C, Supporting Information). Transcriptional analysis revealed upregulation of phagocytosis‐related genes, *mertk*, *mfge8*, and *nr1h3* in indirubin‐treated BMDMs (Figure 5E), confirming enhanced phagocytic activity. Additionally, surface expression of major histocompatibility complex (MHCII) on BMDMs increased in a time‐dependent manner during indirubin incubation (Figure 5F). Notably, pro‐inflammatory gene expression, including IL‐6 and TNF‐α, remained unchanged after 2 h of indirubin exposure, with effects markedly less potent than those induced by zymosan A (Figure 5G; Figure S3D, Supporting Information). Similarly, hepatic FACs‐sorted F4/80^+^ macrophages showed no significant activation after 24‐h indirubin exposure compared to zymosan A‐treated controls (Figure S3E, Supporting Information). Intriguingly, indirubin‐treated BMDMs exhibited reduced capacity to phagocytose *E. coli* relative to vehicle‐treated cells (Figure 5H), likely due to competition from internalized indirubin aggregates. Collectively, these findings suggest that while indirubin internalization enhances selective phagocytic pathways, it minimally affects core macrophage functions.

### 2.6 Indirubin‐Induced Inflammasome Activation Potentiates Hepatic METs Formation

**Figure 6:**
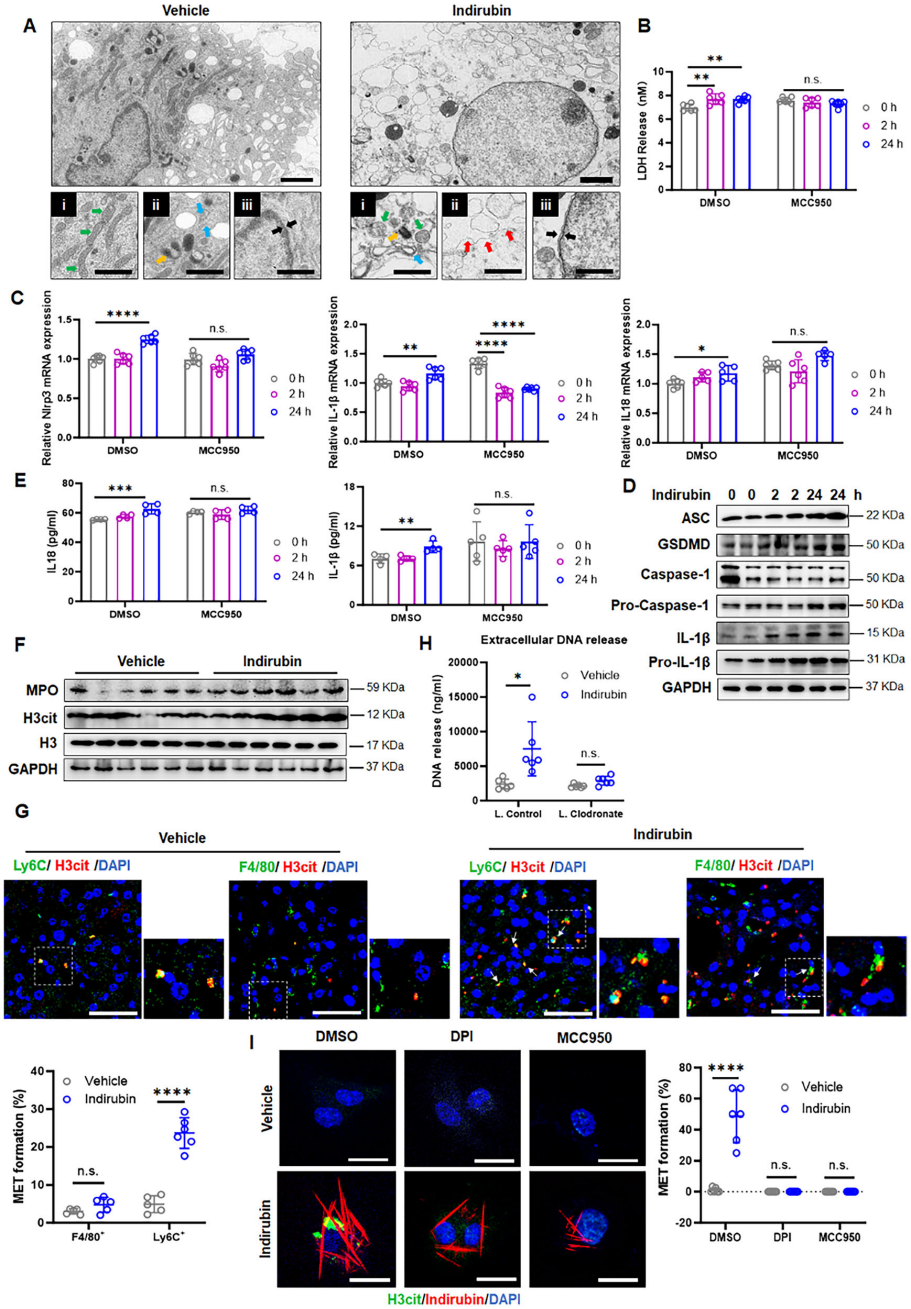
Indirubin‐induced inflammasome activation promotes hepatic METs formation. A) TEM tomography of BMDMs treated with or without indirubin. Subpanels i‐iii indicate mitochondria (green arrowheads), endoplasmic reticulum (blue), Golgi apparatus (orange), plasma membrane ruptures (red), and nuclear membranes (black). Scale bar: 5 µm. B) Lactate dehydrogenase (LDH) levels in supernatants of BMDMs treated with vehicle, indirubin ± MCC950 (NLRP3 inhibitor). Data are expressed as mean ± SD (*n * = 6). C) The mRNA expression of *nlrp3*, *il1β* and *il18*. Data are expressed as mean ± SD (*n * = 6). D) Immunobloting of cleaved caspase‐1, IL‐1β and GSDMD; and E) secreted IL‐1β and IL‐18 protein levels. Data are expressed as mean ± SD (*n * = 4 and 5). F) Hepatic citrullinated histone H3 (H3cit) and MPO protein levels in colitis mice treated with vehicle or indirubin. G) Representative confocal microscopy of hepatic sections stained for DNA (blue), F4/80 (green) and H3Cit (red). METs are marked by white arrows (scale bar: 50 µm). Quantification shows colocalization of extracellular DNA and H3cit. H) Plasma extracellular DNA levels in indirubin‐treated colitis mice ± clodronate‐loaded liposomes. Data are expressed as mean ± SD (*n*  =  6). I) BMDMs stained for DNA (blue) and H3Cit (green) after treatment with indirubin (0.5 µg mL^−1^) ±10 µM DPI (ROS inhibitor) or 10 nM MCC950 for 48 h. Scale bar: 10 µm. METs formation quantification (right panel). n.s., not significant; *p < 0.05; **p < 0.01; ***p < 0.001; ****p < 0.0001 (Student's *t* test or one‐way ANOVA).

Plasma membrane rupture caused by bacteria invasion or crystalline aggregates is a known trigger for NLRP3 inflammasome activation in macrophages.^[^ ^26^ ^]^ Given the observed accumulation of indirubin in the liver of colitis mice, we investigated whether indirubin induces plasma membrane breakdown and NLRP3 inflammasome activation in macrophages in vitro. While BMDMs treated with 0.5 µg mL^−1^ indirubin remained highly viable, they displayed hallmark features of plasma membrane rupture, including organelle swelling and cytoplasmic content leakage (**Figure** **6**A). This was corroborated by increased lactate dehydrogenase (LDH) release, indicative of lytic pore formation (Figure 6B). Additionally, the expression of NLRP3 inflammasome‐related genes, including *nlrp3*, *il‐1β*, and *il‐18* mRNA transcripts, was upregulated in BMDMs following 24 h of indirubin treatment. These changes were reversed by co‐treatment with MCC950, a selective NLRP3 inhibitor (Figure 6C). Immunoblotting confirmed increased levels of activated caspase‐1, mature IL‐1β, and cleaved gasdermin D (GSDMD), alongside elevated secretion of IL‐1β and IL18 proteins (Figure 6D,E). NLRP3 inflammasome activation, resulting from plasma membrane rupture, promotes macrophage lytic death – a process that may facilitate bacterial dissemination or crystal propagation.^[^ ^27^ ^]^ In vivo, hepatic tissues from indirubin‐treated colitis mice exhibited extracellular deposition of citrullinated histone H3 (H3Cit) and myeloperoxidase (MPO), markers of macrophage extracellular traps (METs) (Figure 6F). H3Cit^+^ extracellular traps showed stronger co‐localization with Ly6C^+^ infiltrating macrophages than with resident F4/80^+^ macrophages (Figure 6G), indicating indirubin specifically induces METs formation in recruited hepatic macrophages. Notably, no METs‐associated H3Cit or MPO deposition was detected in pulmonary tissues across treatment groups (Figure S4A,B, Supporting Information), demonstrating indirubin's lack of effect on alveolar or infiltrating pulmonary macrophages.

Furthermore, macrophage‐depleted mice showed significantly reduced extracellular DNA release compared to indirubin‐treated mice without macrophage depletion (Figure 6H). Consistent with this in vivo observation, indirubin‐treated BMDMs exhibited METs formation in vitro. Co‐treatment with the ROS inhibitor, DPI, and MCC950 suppressed this effect (Figure 6I), demonstrating that indirubin‐induced METs formation depends on ROS‐dependent NLRP3 inflammasome activation. Taken together, these data demonstrate that indirubin phagocytosis mechanistically triggers plasma membrane rupture, leading to subsequent NLRP3 inflammasome activation and METs formation.

### 2.7 Indirubin‐Induced METs Exacerbate Oxidative Stress‐Associated Hepatocyte Injury

**Figure 7:**
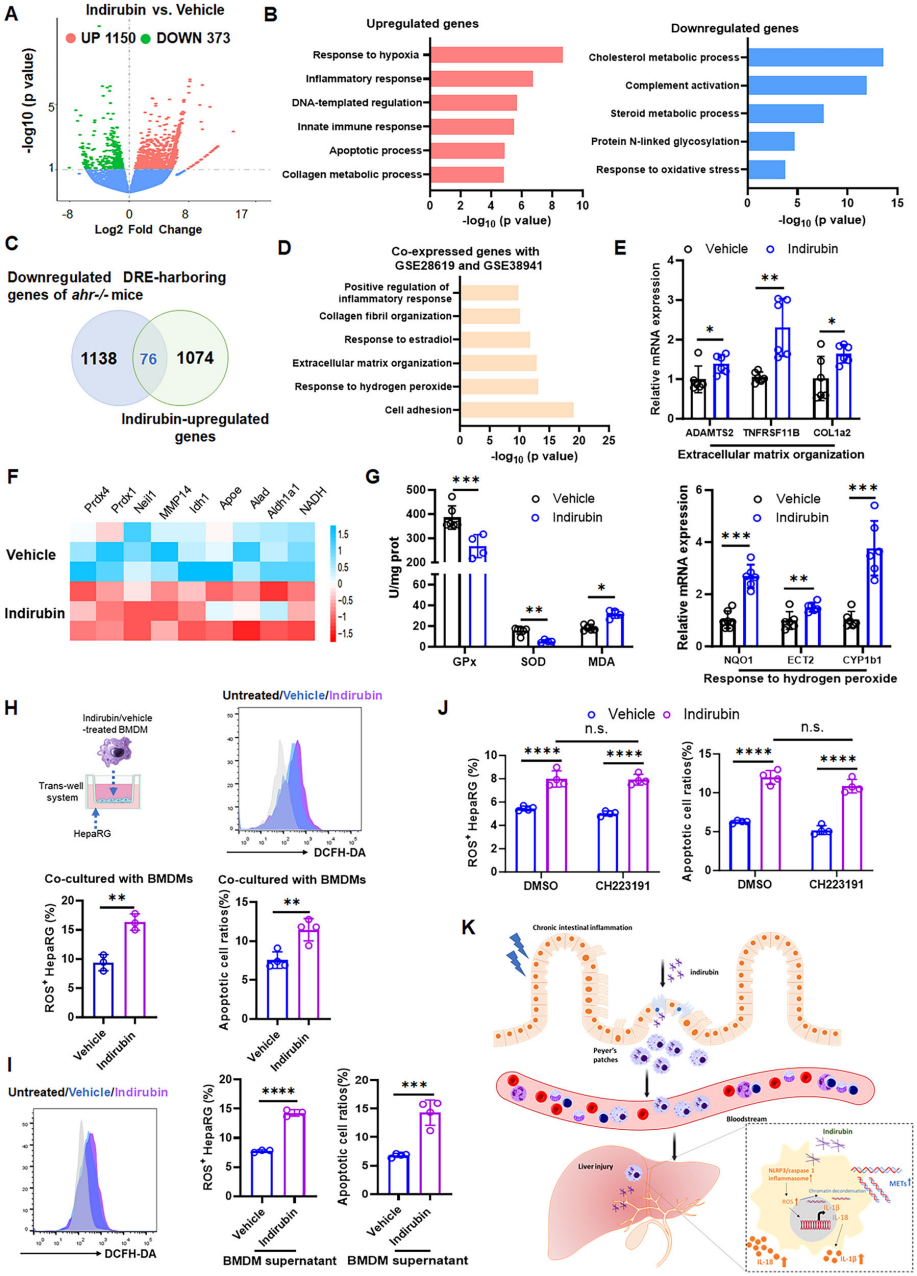
Indirubin‐induced METs exacerbate oxidative stress‐associated hepatocyte injury. A) Volcano plots showing adjusted *p*‐value for differentially expressed genes (DEGs) in vehicle‐ and indirubin‐treated mice. B) Gene ontology (GO) analysis of the DEGs in livers of indirubin‐treated mice. C) Overlap analysis of DEGs between *AhR^−/‐^* mice (genes with DRE binding sites) and indirubin‐treated colitis mice. D) GO analysis of co‐expressed genes in indirubin‐treated colitis mice and human datasets (alcoholic hepatitis: GSE28619; acute liver failure: GSE38941). E) The mRNA expression of extracellular matrix organization‐ and hydrogen peroxide response‐related genes. Data are expressed as mean ± SD (*n*  = 6). F) Oxidative stress response‐related gene expression changes in vehicle‐ and indirubin‐treated mice. G) Hepatic glutathione peroxidase (GPx), superoxide dismutase (SOD) and malondialdehyde (MDA) levels. Data are expressed as mean ± SD (*n*  = 6). H) ROS production and apoptosis (PI/Annexin V assay) in HepaRG cells co‐cultured with indirubin or vehicle pre‐treated BMDMs for 48 h. Data are expressed as mean ± SD (*n*  =  4). I) ROS production and apoptosis in HepaRG incubated with supernatant from vehicle‐ or indirubin‐treated BMDMs for 48 h. Data are expressed as mean ± SD (*n*  = 4). J) ROS production and apoptosis in HepaRG cells directly treated with indirubin or vehicle for 48 h. Data are expressed as mean ± SD (*n*  = 4). K) Proposed mechanism of indirubin aggregates from Peyer's patches are internalised by macrophages, transported to the livers via ciculation, and activate the NLRP3 inflammasome. This triggers METs release, amplifying oxidative stress‐induced liver injury. n.s., not significant; *p < 0.05; **p < 0.01; ***p < 0.001; ****p < 0.0001 (Student's *t* test or one‐way ANOVA).

To elucidate the mechanism of indirubin‐induced hepatocyte injury in chronic colitis, we performed bulk RNA sequencing on hepatic tissues from colitis mice treated with a sublethal indirubin dose. Significant transcriptional changes were identified (*p *< 0.05), with most transcripts being up‐regulated (log_2_ fold change > 1). Specifically, we found that 373 genes were downregulated and 1150 genes were upregulated in the hepatic tissues of indirubin‐treated mice compared to controls (**Figure** **7**A). Gene ontology biological process (GOBP) enrichment analysis of the DEGs indicated that indirubin treatment significantly downregulated metabolic pathways, particularly those involved in cholesterol metabolism and steroid hormone biosynthesis. Conversely, biological processes such as the response to hypoxia, inflammatory response, innate immune response, and apoptotic process were notably upregulated (Figure 7B). Additionally, the minimal expression of hepatic TLRs suggests that endotoxemia was absent in indirubin‐treated mice (Figure S2C, Supporting Information). Analysis of *AhR^−/‐^* mice using the AhR‐binding element (dioxin‐responsive element, DRE) database^[^ ^28^ ^]^ revealed that 1214 DRE‐harboring genes were downregulated in these mice. A comparison between indirubin‐upregulated transcriptomes and the downregulated DRE‐harboring genes indicated that only 76 transcripts overlapped, confirming that the hepatic response to indirubin does not reflect a classic AhR agonist mechanism (Figure 7C; Table S1, Supporting Information). Furthermore, comparative analysis of indirubin‐upregulated genes with gene expression changes in hepatic tissues from patients with alcoholic hepatitis (GSE28619) and acute liver failure (GSE38941) showed that the top enriched co‐expressed pathways were related to extracellular matrix organization, response to hydrogen peroxide, and regulation of the inflammatory response (Figure 7D). Consistently, co‐expressed genes involved in extracellular matrix organization, such as *adamts2*, *tnfrsf11b*, *col1a2*, and hydrogen peroxide response, such as *nqo1*, *ect2*, *cyp1b1*, were significantly increased in indirubin‐treated liver tissues (Figure 7E). Notably, indirubin treatment downregulated oxidative stress response genes, including *pdx1* and *prdx4* (Figure 7F), suggesting enhanced hepatic oxidative stress. Consistent with this, we observed reduced levels of hepatic redox enzymes, including mitochondrial superoxide dismutase (SOD) and cytoplasmic glutathione peroxidase (GPX), alongside elevated malondialdehyde (MDA) in indirubin‐treated mice (Figure 7G).

To further examine whether indirubin‐induced METs potentiate oxidative damage in hepatocytes, we co‐cultured indirubin‐pretreated BMDMs with HepaRG cells in vitro. Hepatocytes co‐cultured with indirubin‐treated BMDMs or incubated with supernatant derived from indirubin‐treated BMDMs exhibited increased ROS production and apoptosis (Figure 7H,I), indicating that indirubin‐induced METs promote hepatocyte injury. Direct treatment of HepaRG cells with indirubin alone also increased ROS production and cell death. However, co‐treatment with the AhR antagonist CH223191 failed to reverse these effects, demonstrating that hepatocyte damage is AhR‐independent (Figure 7J). Collectively, these findings suggest that both indirubin‐derived METs and indirubin itself induce oxidative stress, leading to hepatic injury.

## 3 Discussion

Previous studies demonstrate that indirubin undergoes partial absorption and hepatic metabolism via the first‐pass effect when administered through non‐oral routes (e.g., intravenous or intraperitoneal).^[^ ^29^ ^]^ However, this contrasts with clinical observations, where oral indirubin exhibits extremely low bioavailability due to poor aqueous solubility and rapid systemic metabolism, rendering first‐pass effects clinically insignificant. Early in vitro studies confirmed that hepatic cytochrome P450 enzymes, particularly CYP1A1/2, metabolize indirubin.^[^ ^30^ ^]^ Notably, indirubin also induces CYP enzymes, accelerating its own metabolism – a self‐limiting mechanism that further reduces systemic exposure. While indirubin may undergo phase I metabolism, its poor solubility, and tendency to aggregate likely restrict intestinal absorption and enzymatic access, limiting systemic exposure. Recent studies indicate indirubin modulates gut microbiota by increasing the relative abundance of *Alloprevotella*, *Rikenella*, and *Lactobacillus*,^[^ ^31^, ^32^ ^]^ suggesting intestinal retention of indirubin, though direct evidence of bacterial metabolism remains lacking. Our study detected 10%–15% of unmetabolized indirubin in stool, implying potential bacterial utilization requiring further validation. Indirubin absorption occurs primarily via passive diffusion, with no involvement of P‐glycoprotein or multidrug resistance‐associated protein.^[^ ^33^ ^]^ To date, no conclusive evidence exists for indirubin metabolism by hepatic enzymes or gut bacteria. Our work demonstrates that indirubin, a poorly soluble plant‐derived molecule, induces hepatotoxicity in mice with chronic colitis. This occurs primarily through macrophage adsorption in the Peyer's patches, followed by transport to the liver and lungs. Macrophage phagocytosis of indirubin triggers NLRP3 inflammasome activation and subsequent METs formation (Figure 7K). These results suggest a novel transport mechanism for poorly soluble molecules via macrophages‐mediated circulatory delivery to the liver. To our knowledge, this is the first report of systemic dissemination of an insoluble molecule through macrophage transport.

As key mediators of host defense, macrophages ingest diverse foreign particles, including microbial pathogens, therapeutic agents, and environmental contaminants, for intracellular degradation.^[^ ^34^, ^35^, ^36^ ^]^ Unlike intestinal macrophages, those in Peyer's patches lack anti‐inflammatory characteristics, as their primary role is to neutralize foreign particles and initiate adaptive immune responses.^[^ ^20^ ^]^ Pharmacokinetics studies revealed indirubin distribution in Peyer's patches, plasma, liver, and lung. Further findings – 1) colocalization with CX_3_CR1^+^ macrophages in Peyer's patches and 2) reversal of indirubin's detrimental effects upon macrophage depletion – align with our LC‐MS/MS data indicating macrophage‐mediated transport of indirubin from Peyer's patches into systemic circulation.

Interestingly, while we observed greater indirubin accumulation in the lungs compared to the liver, this did not correlate with noticeable tissue damage. This organ‐specific discrepancy may reflect the differential roles of METs in tissue homeostasis. Hepatic METs are well‐documented to exacerbate iron overload‐related liver ischemia/reperfusion injury via ferroptosis.^[^ ^37^ ^]^ Consistently, exposure to polystyrene microplastics caused liver fibrotic injury in mice, characterized by macrophage recruitment and METs formation,^[^ ^38^ ^]^ whereas emerging evidence indicates that pulmonary METs may serve protective roles – for instance, enhancing phagocytic clearance of particulate matter without causing airway injury.^[^ ^39^ ^]^ Our findings align with this dichotomy, as indirubin accumulation in the lungs did not induce MET‐associated injury. Clinically, pulmonary arterial hypertension, but not alveolar injury is the reported adverse effect of indirubin, implicating vascular dysfunction (e.g., endothelial cell targeting) rather than parenchymal damage. This supports our observation that lung accumulation does not equate to alveolar toxicity. Further studies on lung‐specific transporters or endothelial pathways are warranted to unravel indirubin's pulmonary pharmacokinetics.

Indirubin and indigo are potent ligands of AHR, with indirubin being a 50‐fold more potent agonist than indigo.^[^ ^40^ ^]^ When comparing altered transcriptomes with those induced by tetrachlorodibenzo‐p‐dioxin (TCDD), a potent AHR agonist, we observed only 76 overlapping differentially expressed genes. While we cannot preclude the potential AhR‐mediated effects of released indirubin on hepatic cells, our in vitro study demonstrated that direct indirubin exposure damages hepatocytes through an AhR‐independent mechanism, as evidenced by the inability of the AhR antagonist CH223191 to reverse these effects. Importantly, our current study revealed that indirubin‐internalizing macrophages exhibit ruptured plasma membrane, NLRP3 inflammasome activation, and subsequent METs formation. METs are specialized structures produced during liver injury to eliminate invading foreign particles in response to chemical stimuli and cytokines.^[^ ^38^, ^41^ ^]^ This process, termed ETosis, is a unique form of programmed cell death characterized by the release of extracellular traps (ETs) – DNA‐protein complexes expelled from immune cells to immobilize pathogens. Initially described in neutrophils as NETosis, ETosis has since been extended to other immune cells, including macrophages (METosis) and eosinophils (EETosis).^[^ ^42^ ^]^ Unlike apoptosis (marked by cytoplasmic shrinkage and apoptotic body formation)^[^ ^43^ ^]^ or autophagy (defined by autophagosome‐mediated cytoplasmic vacuolization),^[^ ^44^ ^]^ ETosis involves nuclear disintegration and expulsion of chromatin mixed with antimicrobial proteins to form extracellular “traps”,^[^ ^45^ ^]^ ultimately leading to cell death. Transcriptomic analysis further confirmed oxidative stress as the primary regulatory mechanism in the livers of indirubin‐treated mice. These findings suggest that indirubin‐induced METs exacerbate oxidative damage in hepatic tissue.

Given that most herbal compounds, such as indirubin, are poorly water‐soluble, our findings offer valuable insights for optimizing clinical formulations to improve safety. For instance, designing “bio‐invisible” nanomaterials for indirubin encapsulation, such as nanoparticles surface‐functionalized with CD47 (“don't eat me”) ligands to evade macrophage recognition via signal regulatory protein (SIRP)‐α binding, could reduce hepatic uptake.^[^ ^34^ ^]^ Studies indicate that macrophages preferentially internalize larger particles (1–3 µm in size),^[^ ^46^ ^]^ while smaller particles (< 500 nm) exhibit higher rates of exocytosis.^[^ ^47^ ^]^ Alternatively, an enteric‐coated micro‐pellet formulation could enable targeted intestinal delivery, minimizing systemic distribution and macrophage uptake. Future studies should focus on optimizing indirubin‐loaded nanomaterials or particle size to balance intestinal retention with avoidance of phagocytic clearance. Beyond formulation strategies, combining indirubin with anti‐METosis therapies could reduce the risk of METs‐induced liver injury. For instance, lactoferrin, a glycoprotein known to suppress NETs, has been shown to decrease METs generation and alleviate acute kidney injury in murine models of rhabdomyolysis‐induced renal damage.^[^ ^27^ ^]^ Similarly, DNase 1 and α1‐antitrypsin mitigate lung inflammation by degrading METs and neutralizing proteases, respectively, a therapeutic approach under investigation for cystic fibrosis lung disease.^[^ ^48^ ^]^ Conversely, cigarette smoke exacerbates lung damage in chronic obstructive pulmonary disease by increasing METs production; DNase treatment may counteract this by destroying METs and restoring protease balance.^[^ ^49^ ^]^ Beyond direct METs degradation, inhibiting PAD2 and PAD4 (enzymes critical for synthesizing citrullinated METs) has shown promise in treating rheumatoid arthritis.^[^ ^50^ ^]^ These approaches collectively aim to reduce inflammation and tissue damage linked to METosis. Integrating indirubin with anti‐METosis therapies may enable safer treatment regimens that mitigate METs‐related complications.

In conclusion, our study demonstrates that indirubin, the major bioactive molecule of indigo naturalis, contributes to hepatic impairment in mice with chronic colitis. The mechanism does not involve the breakdown of the gut‐blood barrier and passive crossing, as proposed for bacteria translocation.^[^ ^51^ ^]^ Instead, it is mediated by phagocytosis of indirubin aggregates by pro‐inflammatory macrophages in Peyer's patches. Our study posits a novel mechanism whereby indirubin bypasses traditional absorption routes and is absorbed via the intestinal lymphatic system, potentially explaining its paradoxical hepatic toxicity. This lymphatic route could direct indirubin to the liver without first‐pass metabolism. Our findings highlight a transport pathway with significant promise for targeted drug delivery, particularly in liver diseases such as cirrhosis and hepatocellular carcinoma. Poorly soluble pharmacological agents could be engineered to exploit Peyer's patches, enabling direct hepatic delivery and increasing local drug concentrations. Moreover, this mechanism may extend to other water‐insoluble, plant‐derived therapeutics, such as curcumin. While Peyer's patches allow particles to bypass gastrointestinal absorption barriers and first‐pass metabolism, this route risks unintended hepatic accumulation. Clinical success will require meticulous optimization of formulation parameters (e.g., particle size, shape, and surface coating) and dosage regimens, with deliberate consideration of this delivery mechanism. Rigorous dose monitoring and tissue‐specific targeting strategies are essential to enhance delivery efficiency to target cells while minimizing off‐tissue effects, thereby improving therapeutic outcomes and reducing systemic toxicity.

## 4 Experimental Section

### Chemicals and Drugs

Indigo Naturalis (IN) was purchased from Sichuan Qianfang Pharmaceutical Co., Ltd. (Chengdu, China). HPLC analysis confirmed that IN contains 2.38% indigo (ING) and 0.39% indirubin (INB). Reference standards of indigo (ING) (A0264; ≥98% purity) and indirubin (INB) (A1147; ≥98% purity) were obtained from Chengdu Must Bio‐technology Co., Ltd. (Chengdu, China).

### Mice

C57BL/6J wild‐type mice (7–8 weeks old, 21–24 g) were purchased from The Chinese University of Hong Kong (China). *IL‐10^1Cgn^*/J mice (6–8 weeks old, 21–25 g) were acquired from The Jackson Laboratory (Maine, USA). Mice were housed under a 12‐h light‐dark cycle with ad libitum access to food and water. Euthanasia was performed via cervical dislocation under anesthesia or anesthetic overdose. All procedures were approved by the Committee on the Use of Human and Animal Subjects in Teaching and Research (HASC) of Hong Kong Baptist University (Protocol No. REC/19‐20/0301) and complied with the Animals Ordinance of the Department of Health, Hong Kong SAR, China.

### Model Establishment

Chronic colitis was induced in C57BL/6J mice using three cycles of dextran sulfate sodium (DSS) administration. Each cycle comprised 1 week of 1.8% (w/v) DSS (MP Biomedicals, Aurora, USA) in drinking water followed by 1 week of recovery. The mice were randomized into four groups: 1) the control group received 200 µL of sterile water orally each day; 2 and 3) the ING or INB groups were given 1 g kg day^−1^ of ING or INB, respectively, each suspended in an equal volume of sterile water; 4) the IN group was administered 6 g kg day^−1^ of IN orally. For FITC‐labeled particle tracking, insoluble artificial particles (5–7.9 µm; Spherotech, FP‐6052‐2) were labeled with fluorescein isothiocyanate (FITC) and administered orally (1 g kg^−1^). Tissue distribution was monitored at 2 and 6 h post‐administration using an IVIS imaging system. Liver tissues were embedded in an OCT compound, sectioned (8 µm thickness), and imaged via STED confocal microscopy.

### Hepatic Transaminases Measurement

Serum alanine aminotransferase (ALT) and aspartate aminotransferase (AST) levels were quantified using Stanbio Laboratory kits (#2930 and #2920, respectively) per manufacturer protocols.

### TUNEL Assay

Liver tissues were fixed in 4% paraformaldehyde. Apoptotic cells in paraffin‐embedded sections were detected using the TUNEL Assay Kit – HRP‐DAB (ab206386, Abcam, USA) or Click‐iT Plus TUNEL Assay (C10617, Invitrogen, USA). Fluorescence microscopy (Leica, Germany) was used for imaging, with TUNEL‐positive cells quantified by three independent investigators.

### Masson's Trichrome Staining

Collagen deposition was assessed using a trichrome stain kit (ab150686, Abcam, USA). After deparaffinization and hydration in distilled water, tissue sections were immersed in pre‐warmed Bouin's fluid (56–64 °C) for 60 min, followed by cooling for 10 min. Sections were rinsed thoroughly with water until clear, then treated with Weigert's iron hematoxylin for 5 min. After rinsing, Biebrich scarlet/acid fuchsin solution was applied for 15 min. Sections were treated with phosphomolybdic/phosphotungstic acid until collagen destaining occurred, followed by aniline blue solution (10 min), a rinse, and 1% acetic acid treatment (5 min). Finally, sections were dehydrated in 95% ethanol (two rounds) and visualized under a microscope.

### 16S rRNA FISH

Tissue sections were hybridized overnight at 37 °C with Cy3‐labeled eubacterial probes (MBD0033, Sigma, USA). Sections were incubated at 48 °C for 15 min the next day, rinsed in pre‐cooled water, counterstained with DAPI, and imaged using a confocal microscope.

### Immunohistochemical (IHC) Staining

Formalin‐fixed, paraffin‐embedded (FFPE) liver sections were deparaffinized in xylene, rehydrated through graded ethanol, and subjected to antigen retrieval in citrate buffer (pH 6.0). Endogenous peroxidase activity was blocked with 0.3% hydrogen peroxide in PBS (10 min). Sections were incubated overnight with primary antibodies, washed three times with PBS, and incubated with secondary IgG and DAPI. Slides were mounted with fluorescent anti‐fade medium and analyzed by confocal microscopy.

### LC‐MS/MS Quantification

Tissues were homogenized in dimethylformamide (DMF). Plasma samples were dried under nitrogen and reconstituted in 200 µL DMF. After centrifugation (12 000 rpm, 10 min), indirubin levels were analyzed using an Agilent 1290 Infinity II – 6470 LC/TQ system (USA) with a ZORBAX Eclipse Plus C18 column (2.1 × 100 mm, 1.8 µm; 40 °C). The mobile phase comprised 0.1% formic acid in water (A) and acetonitrile (B), with a gradient from 5% to 66% B over 8 min (flow rate: 0.4 mL min^−1^; injection volume: 1 µL). Indirubin was detected via MRM (ESI+, m/z 263→219) with the following parameters: gas temperature 350 °C, drying gas 10 L min^−1^, nebulizer 45 psi, sheath gas 350 °C, sheath flow 8 L min^−1^, capillary voltage 4.0 kV.

### Isolation of Single‐Cell Suspensions from Peyer's Patches

Tissues were dissociated using a syringe plunger, digested in RPMI‐1640 containing collagenase IV and DNase I (Sigma, USA; 37 °C, 30 min), vortexed (15 sec ), filtered through a 70‐µm strainer, and centrifuged (400 × g, 5 min). Cells were resuspended in PBS for analysis.

### Peripheral Blood Mononuclear Cell (PBMC) Isolation

Whole blood was stained with monoclonal antibodies (20 min, 4 °C), treated with ACK lysis buffer (A1049201, Thermo Fisher, USA), centrifuged, and resuspended in PBS.

### Macrophage Depletion

Clodronate‐loaded liposomes (LIPOSOMA BV, Netherlands) were administered intraperitoneally (100 µL of 5 mg mL^−1^ per mouse, twice weekly, days 16–40) to chronic colitis mice ± indirubin. Clodronate induces macrophage apoptosis via liposome internalization and intracellular accumulation.^[^ ^52^, ^53^ ^]^ Depletion was confirmed by macrophage‐specific staining (Figure 4F).

### Bone Marrow‐Derived Macrophage (BMDM) Culture

Bone marrow from C57BL/6J mouse tibias/femurs was cultured in RPMI‐1640 with 10 ng mL^−1^ M‐CSF (PMC2044, Thermo Fisher, USA). On day 7, cells were stimulated with LPS (250 ng mL^−1^; L8880, Solarbio, China) and IFN‐γ (100 ng mL^−1^; 575 306, BioLegend, USA) for 24 h.

### RT‐PCR

RNA was extracted using TRIzol, resuspended in RNA‐free water (100 µL), and reverse‐transcribed (5 µg RNA) with a SuperScript kit (Invitrogen). SYBR Green Master Mix was used for amplification.

### Phagocytosis Assay

BMDMs (1 × 10^5 ^cells) ± INB were incubated with pHrodo Green *E. coli* BioParticles (P35366, Invitrogen; 37 °C, 1 h). Phagocytosis was quantified by flow cytometry (pHrodo Green‐positive cells).

### Transcriptomic Analysis

Liver RNA was extracted using RNeasy Plus Micro Kit (Qiagen, Germany). Libraries (400 ng RNA) were sequenced on a DNBSEQ platform (BGI), aligned to the mouse genome, and analyzed.

### ROS Staining

Cells were washed (PBS ×3), incubated with H2DCFDA (D399, Invitrogen; 37 °C, 20 min), stained with Hoechst 33342 (#14533, Sigma), and imaged. ROS levels (DCFH fluorescence) were quantified by flow cytometry (FACS Celesta, BD Biosciences; 488/525 nm).

### Annexin V/PI Apoptosis Detection

Apoptosis was assessed using an Annexin V/PI detection kit (556547, BD Pharmingen) per the manufacturer's protocol. Briefly, 1 × 10^4^ hepatic cells were trypsinized post‐induction, washed twice in ice‐cold PBS, and centrifuged (800 × g, 3 min). Cells were resuspended in 200 µL binding buffer and incubated with 3 µL Annexin V‐FITC and 3 µL PI (20 min, room temperature, dark). Samples were immediately analyzed using a FACS Celesta flow cytometer (BD Biosciences).

### Oxidative Stress Markers

Superoxide dismutase (SOD), catalase (CAT), and glutathione peroxidase (GPX) activities in liver homogenates were measured using Solarbio kits (China). Lipid peroxidation was quantified via malondialdehyde (MDA) levels using a thiobarbituric acid‐reactive substances (TBARS) assay (MDA Assay Kit #BC0025, Solarbio, China).

### Western Blot

Liver proteins were extracted with RIPA lysis buffer (R0010, Solarbio, China) and quantified via BCA assay (Bio‐Rad, USA). Proteins (8–10 µg) were separated on 10% SDS‐PAGE gels, transferred to PVDF membranes, and incubated with 5% BSA (w/v) for 1 h. Membranes were probed with primary antibodies (4 °C, overnight), followed by HRP‐conjugated secondary antibodies (2 h, room temperature). Blots were visualized using an ECL kit and imaged (Thermo Scientific, USA).

### TEM Sample Preparation

Cells (1 × 10^6^) were collected in a growth medium using a cell scraper, centrifuged (300–500 × g, 5–10 min), and fixed in 2.5% glutaraldehyde (1 h, room temperature). After PBS washes (×3), samples were post‐fixed in 1% OsO4 (1–2 h), rinsed in 0.1 M PBS (×3), dehydrated, and embedded. Polymerization occurred at 70 °C (12–48 h). Ultrathin sections (70–90 nm) were cut with an ultramicrotome, mounted on copper grids, and imaged immediately using a Hitachi HT780 TEM.

### Immunofluorescence (IF)

BMDMs were fixed in 4% paraformaldehyde, blocked with 3% BSA, and incubated overnight with anti‐H3cit antibody (ab5103, Abcam) and secondary antibody. Coverslips were mounted with ProLong Gold anti‐fade reagent and imaged via STED confocal microscopy.

### Enzyme‐Linked Immunosorbent Assay, ELISA

BMDM supernatants were added to IL‐1β/IL‐18 antibody‐coated microwells. After washing, peroxidase‐conjugated detection antibodies were added. Substrate‐chromogen mix was incubated, and reactions were stopped with an acidic solution. Optical density (450 nm) was measured using a microplate reader. Cytokine concentrations (pg/mL) were calculated against standard curves.

### Statistical Analysis

Data are expressed as mean ± standard error of the mean (SEM) or mean ± standard deviation (SD) after at least three separate trials. Statistical analyses were performed using the GraphPad Prism (version 8.2, USA). A two‐sided unpaired or paired Student's *t*‐test was used for comparisons between the two groups. Statistical differences among different groups were evaluated using either one‐way or two‐way analysis of variance (ANOVA). A *p* < 0.05 threshold was applied.

## Conflict of Interest

The authors declare no conflict of interest.

## Author Contributions

Z.X.B. and H.Y.T. conceived and designed the study; Y.X., J.S., and H.L.M. did the experiments; C.L., J.C., and C.H. facilitated animal experiments; H.Q. and C.L. provided essential insight on the study; Y.X. and H.Y.T. interpreted the results and wrote the manuscript. All authors approved the final manuscript.

## Supporting information



Supporting Information

## Data Availability

The raw and processed data of transcriptomics reported in this study is available at the Gene Expression Omnibus under accession number GSE299769. Other data are available in the Supporting Information of this article or from the corresponding author upon request.
