# Supplementary material for: Macrophage‐Mediated Transport of Insoluble Indirubin Induces Hepatic Injury During Intestinal Inflammation
Source: Adv Sci (Weinh). 2025 Jun 23;12(36):e02993. doi: 10.1002/advs.202502993 (PMC12463038; doi:10.1002/advs.202502993)
Supplement: Supplementary file 1 — Supporting Information [file ADVS-12-e02993-s001.docx]

Supporting Information

Macrophage-Mediated Transport of Insoluble Indirubin Induces Hepatic Injury during Intestinal Inflammation

*Yiqi Xu^1^, Jingchun Shi^1^, Heung-Lam Mok^1, 2^, Cheng Lyu^1, 2^, Junbang Chen^1, 2^, Chunhua Huang^1^, Hongyan Qin^1, 3^, Chengyuan Lin^1^, Hor-Yue Tan^1, 2,^ *, and Zhaoxiang Bian^1, 2,^ **


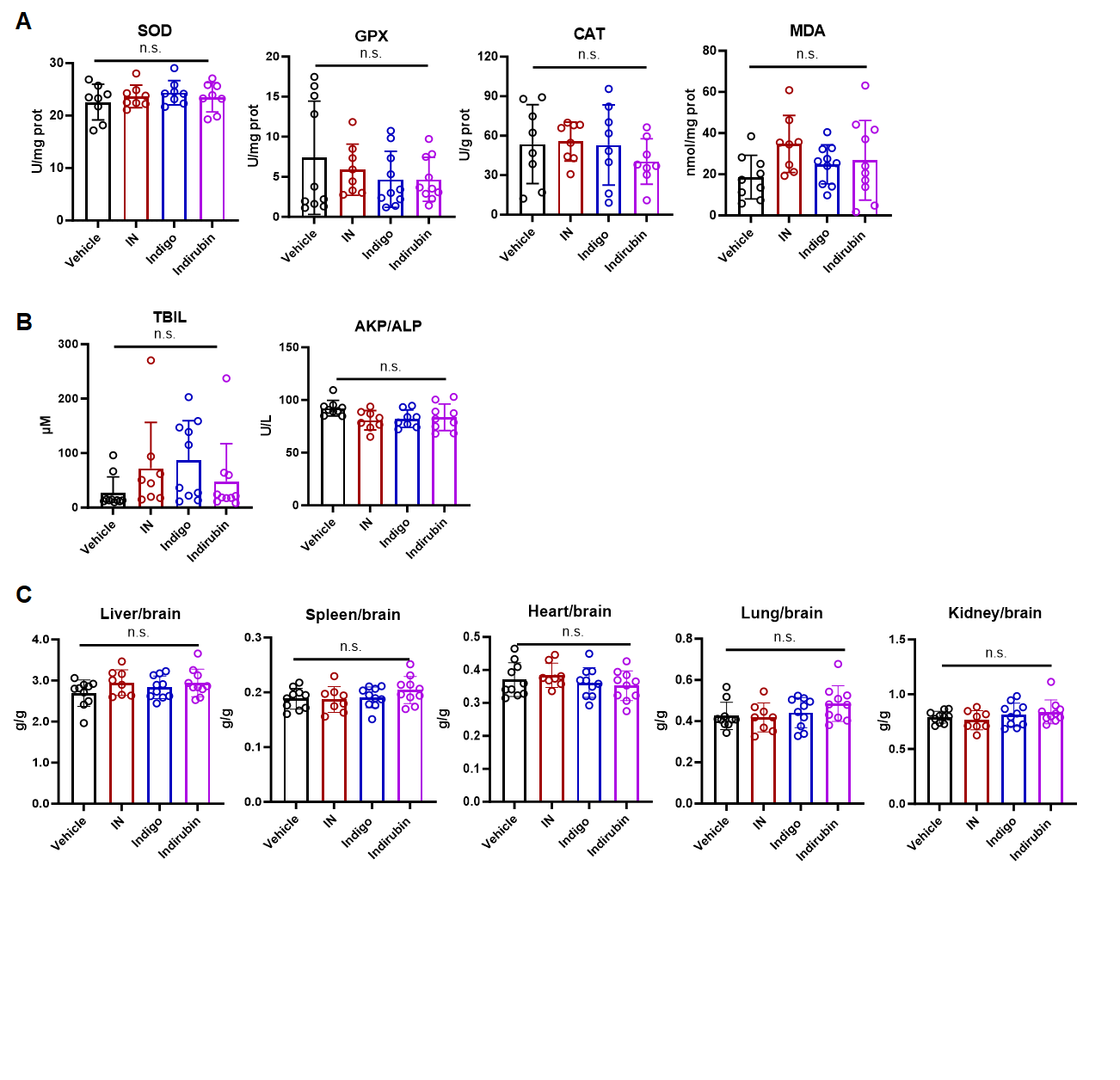


**Supplementary Figure 1. Long-term sub-lethal indirubin administration predisposes colitis mice to hepatic injury.** (A) Hepatic levels of superoxide dismutase (SOD), glutathione peroxidase (GPX), catalase (CAT), and malondialdehyde (MDA) in healthy mice treated with IN, indigo, or indirubin. (B) Serum total bilirubin (TBIL) and alkaline phosphatase (AKP/ALP) levels in healthy mice. (C) Organ weights of liver, spleen, heart, lung, kidney and brain measured at experimental endpoint. Data are expressed as mean ± SD (n = 10 mice/group). n.s., not significant (One-way ANOVA)


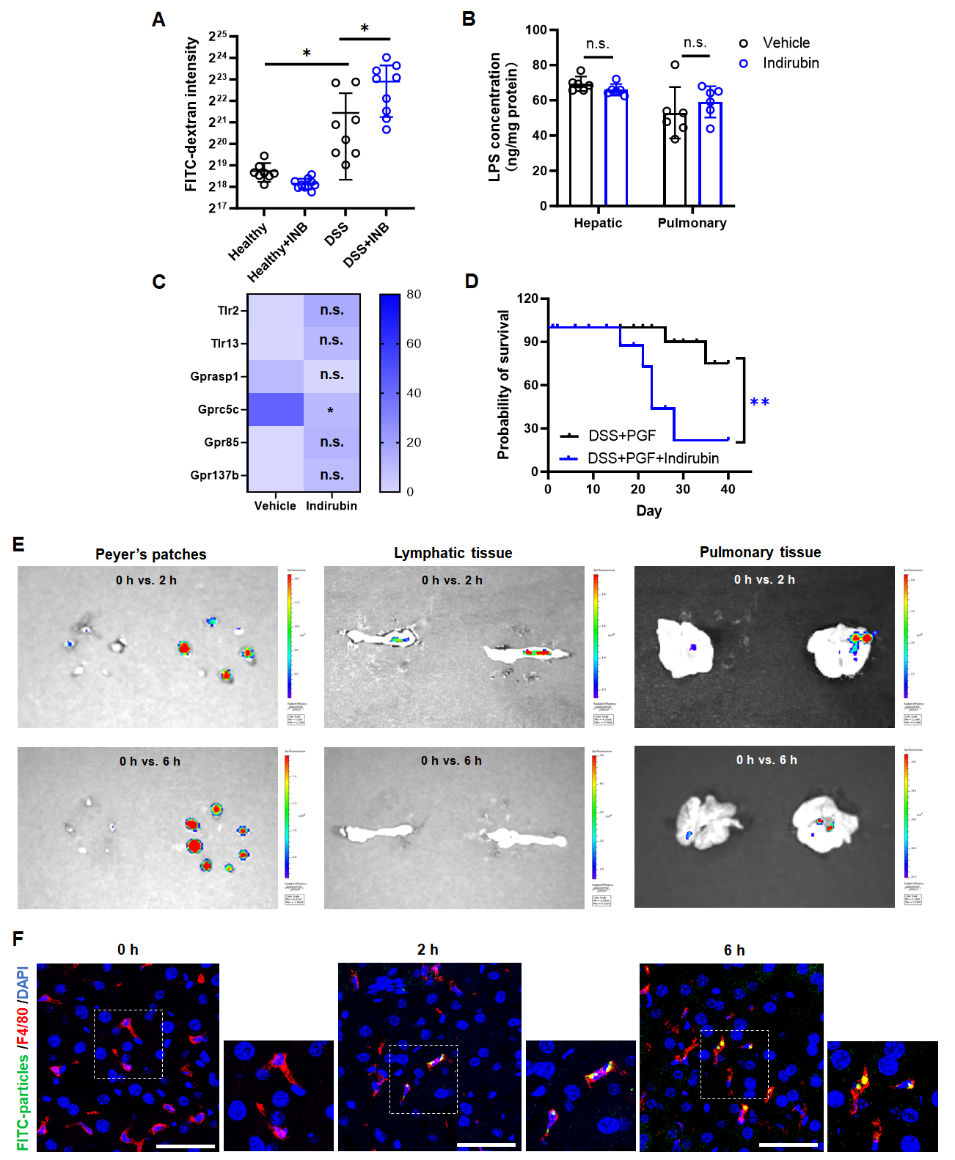


**Supplementary Figure 2. Indirubin accumulation in Peyer’s patches and hepatic tissue during chronic colitis.** (A) Intestinal permeability assessed by serum FITC-dextran (4kDA) fluorescence intensity in vehicle- and indirubin-treated mice. Data are expressed as mean ± SD (n = 10 mice/group). (B) Lipopolysaccharide (LPS) levels in hepatic and pulmonary tissues from colitis mice treated with vehicle or indirubin. (C) Gene expression changes of microbial metabolite receptors in vehicle- versus indirubin-treated mice. (D) Survival analysis of pseudo-germ-free mice treated with vehicle or indirubin. (n = 6 mice/group) (E) In vivo imaging system (IVIS) images of tissues collected at 0, 2 and 6 h following oral administration of FITC-labelled particles. (F) Quantification of FITC-labelled particles in the liver. Mice were euthanized at 0, 2 and 6 h post-administration. Representative immunofluorescence images show nuclear (DAPI, blue), macrophages (F4/80, red) and FITC-labeled particles (green). Scale bar: 50 µm. n. s., not significant; **p*<0.05; ***p*<0.01; (Multiple *t*-test or one-way ANOVA)


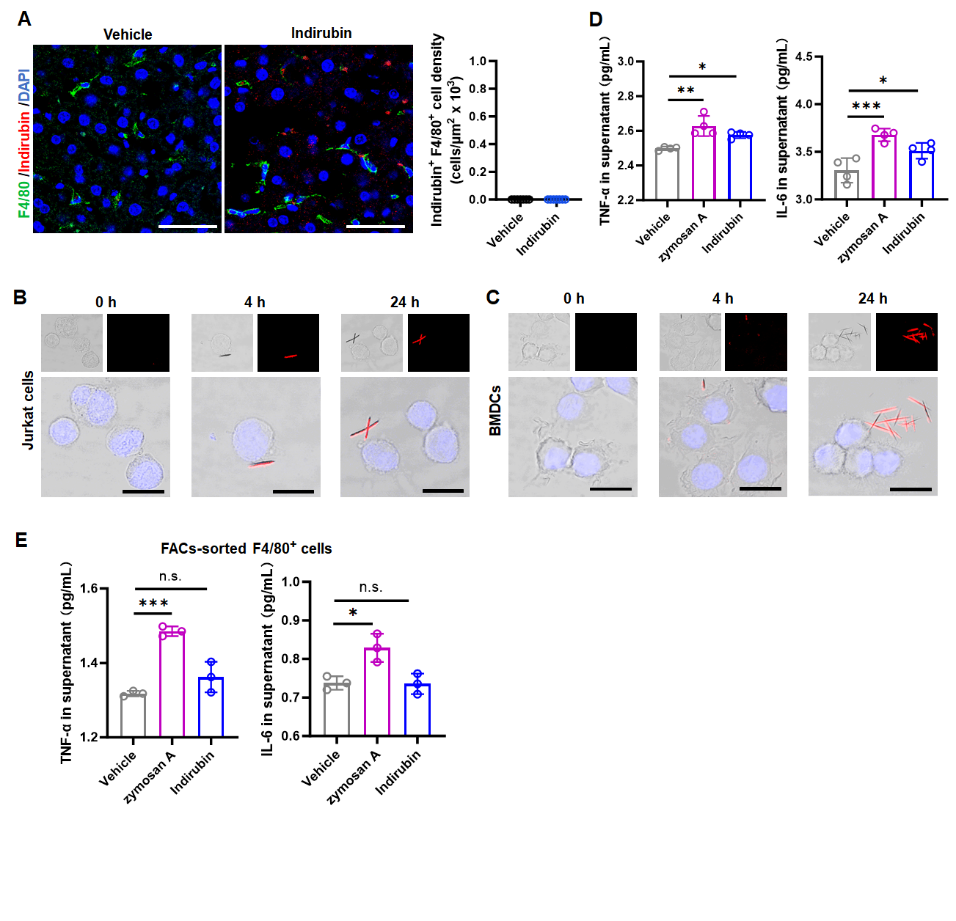


**Supplementary Figure 3. Liver resident macrophages are not activated by indirubin.** (A) F4/80^+^ macrophages in vehicle- and indirubin-treated mice. Scale bar: 50 μm. Phagocytosis of indirubin by (B) Jurkat cells and (C) bone marrow-derived dendritic cells (BMDC) at indicated time points. Scale bar: 10 μm. (D) Inflammatory cytokines levels in culture supernatants of BMDMs treated with indirubin or zymosan A for 24 h. Data are expressed as mean ± SD (n = 4). (E) Inflammatory cytokines in culture supernatants of FACs-sorted hepatic F4/80^+^ cells treated with indirubin for 24 h. Data are expressed as mean ± SD (n = 3). n. s., not significant; **p*<0.05; ***p*<0.01; ****p*<0.001 (Multiple *t* test or one-way ANOVA)


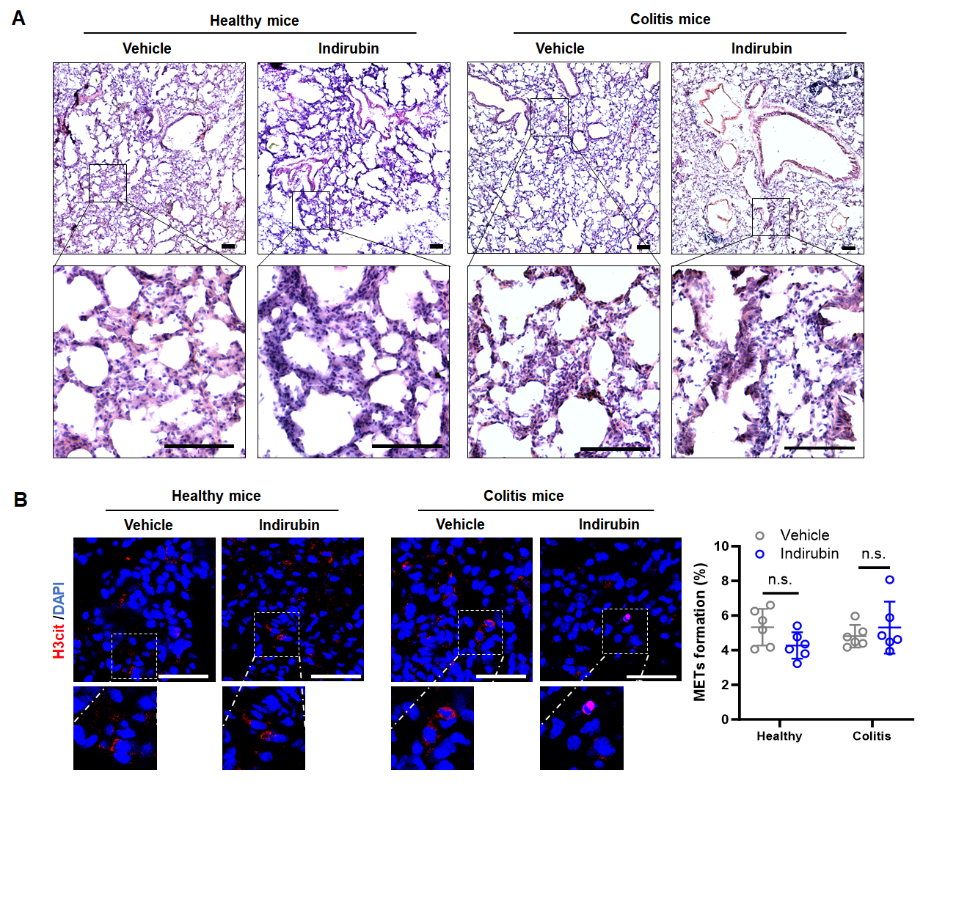


**Supplementary Figure 4. Absence of indirubin-induced macrophage extracellular traps (METs) formation in pulmonary tissue.** (A) H&E staining of lung tissues from healthy and colitis mice ± indirubin treatment. Scale bar: 100 µm. (B) Representative images of H3Cit staining (red) showing METs in lung sections from control and indirubin-treated mice. Scale bar: 50 µm. Quantification of METs-positive areas per field (right panel). Data are expressed as mean ± SD (n = 6). n. s., not significant (Multiple *t*-test or one-way ANOVA)

**Supplementary Table 1. The downregulated DRE-harboring genes**

| gene_id | INB | DSS | log2FoldChange | p_value | p_adj | gene_  name | gene_chr | gene_  length | gene_description |
| --- | --- | --- | --- | --- | --- | --- | --- | --- | --- |
| ENSMUSG00000032315 | 156.1812792 | 0 | 9.703214664 | 0.013015436 | 0.25380101 | Cyp1a1 | 9 | 2787 | cytochrome P450, family 1, subfamily a, polypeptide 1 [Source:MGI Symbol;Acc:MGI:88588] |
| ENSMUSG00000072941 | 200.8904031 | 84.50841606 | 1.254173859 | 0.016046196 | 0.280278261 | Sod3 | 5 | 3738 | superoxide dismutase 3, extracellular [Source:MGI Symbol;Acc:MGI:103181] |
| ENSMUSG00000036905 | 267.0962229 | 95.78201835 | 1.47311299 | 0.003428433 | 0.150728323 | C1qb | 4 | 1088 | complement component 1, q subcomponent, beta polypeptide [Source:MGI Symbol;Acc:MGI:88224] |
| ENSMUSG00000003617 | 4548.899373 | 1781.020432 | 1.353141719 | 0.024701524 | 0.324668094 | Cp | 3 | 9566 | ceruloplasmin [Source:MGI Symbol;Acc:MGI:88476] |
| ENSMUSG00000067336 | 30.36432951 | 6.389298628 | 2.245858014 | 0.047720189 | 0.452296134 | Bmpr2 | 1 | 17983 | bone morphogenetic protein receptor, type II (serine/threonine kinase) [Source:MGI Symbol;Acc:MGI:1095407] |
| ENSMUSG00000003849 | 19.39850279 | 2.26209558 | 3.036036017 | 0.022549501 | 0.312852162 | Nqo1 | 8 | 1553 | NAD(P)H dehydrogenase, quinone 1 [Source:MGI Symbol;Acc:MGI:103187] |
| ENSMUSG00000038530 | 23.19740357 | 2.999436773 | 3.091189353 | 0.032424264 | 0.376115817 | Rgs4 | 1 | 3098 | regulator of G-protein signaling 4 [Source:MGI Symbol;Acc:MGI:108409] |
| ENSMUSG00000022353 | 131.5291144 | 59.22145922 | 1.150630161 | 0.040557954 | 0.41446466 | Mtss1 | 15 | 11345 | MTSS I-BAR domain containing 1 [Source:MGI Symbol;Acc:MGI:2384818] |
| ENSMUSG00000033880 | 215.1631557 | 94.37988258 | 1.190402823 | 0.023642219 | 0.316698471 | Lgals3bp | 11 | 2847 | lectin, galactoside-binding, soluble, 3 binding protein [Source:MGI Symbol;Acc:MGI:99554] |
| ENSMUSG00000024066 | 164.3039264 | 65.99898758 | 1.313245775 | 0.034000616 | 0.383275432 | Xdh | 17 | 9175 | xanthine dehydrogenase [Source:MGI Symbol;Acc:MGI:98973] |
| ENSMUSG00000030401 | 11.65465938 | 0 | 5.966287574 | 0.025010584 | 0.32728371 | Rtn2 | 7 | 4585 | reticulon 2 (Z-band associated protein) [Source:MGI Symbol;Acc:MGI:107612] |
| ENSMUSG00000027074 | 78.94000208 | 25.94346475 | 1.611076651 | 0.02992149 | 0.359511235 | Slc43a3 | 2 | 4464 | solute carrier family 43, member 3 [Source:MGI Symbol;Acc:MGI:1931054] |
| ENSMUSG00000027995 | 16.55677095 | 0.968531959 | 3.960797769 | 0.034627235 | 0.387465809 | Tlr2 | 3 | 3014 | toll-like receptor 2 [Source:MGI Symbol;Acc:MGI:1346060] |
| ENSMUSG00000026600 | 15.19623106 | 1.777829601 | 3.022924792 | 0.042429584 | 0.424924462 | Soat1 | 1 | 9791 | sterol O-acyltransferase 1 [Source:MGI Symbol;Acc:MGI:104665] |
| ENSMUSG00000026390 | 304.8072488 | 116.8871437 | 1.389200091 | 0.033566183 | 0.382442291 | Marco | 1 | 1925 | macrophage receptor with collagenous structure [Source:MGI Symbol;Acc:MGI:1309998] |
| ENSMUSG00000000184 | 43.45690253 | 7.108036888 | 2.551871964 | 0.012104213 | 0.245056007 | Ccnd2 | 6 | 9409 | cyclin D2 [Source:MGI Symbol;Acc:MGI:88314] |
| ENSMUSG00000029084 | 83.78686573 | 32.35168504 | 1.381472548 | 0.028352441 | 0.348761417 | Cd38 | 5 | 3890 | CD38 antigen [Source:MGI Symbol;Acc:MGI:107474] |
| ENSMUSG00000024642 | 25.76222862 | 4.685066234 | 2.402205663 | 0.035155892 | 0.390092662 | Tle4 | 19 | 5702 | transducin-like enhancer of split 4 [Source:MGI Symbol;Acc:MGI:104633] |
| ENSMUSG00000022358 | 18.51408095 | 0 | 6.632013591 | 0.000321807 | 0.0464015 | Fbxo32 | 15 | 6976 | F-box protein 32 [Source:MGI Symbol;Acc:MGI:1914981] |
| ENSMUSG00000020154 | 73.56063097 | 21.46455328 | 1.781740688 | 0.016940086 | 0.287162971 | Ptprb | 10 | 13333 | protein tyrosine phosphatase, receptor type, B [Source:MGI Symbol;Acc:MGI:97809] |
| ENSMUSG00000023913 | 44.66392249 | 4.678727551 | 3.277728539 | 0.013665351 | 0.260557799 | Pla2g7 | 17 | 2905 | phospholipase A2, group VII (platelet-activating factor acetylhydrolase, plasma) [Source:MGI Symbol;Acc:MGI:1351327] |
| ENSMUSG00000024140 | 427.8565307 | 201.5841266 | 1.080263699 | 0.031687561 | 0.372188161 | Epas1 | 17 | 5590 | endothelial PAS domain protein 1 [Source:MGI Symbol;Acc:MGI:109169] |
| ENSMUSG00000024501 | 35.29157665 | 10.38252771 | 1.768033348 | 0.046278503 | 0.44501438 | Dpysl3 | 18 | 6608 | dihydropyrimidinase-like 3 [Source:MGI Symbol;Acc:MGI:1349762] |
| ENSMUSG00000022641 | 30.82973385 | 3.389861855 | 3.086041237 | 0.017465027 | 0.289163775 | Bbx | 16 | 14205 | bobby sox HMG box containing [Source:MGI Symbol;Acc:MGI:1917758] |
| ENSMUSG00000026698 | 15.9970777 | 1.210664948 | 3.571031621 | 0.026389886 | 0.336930045 | Pigc | 1 | 4250 | phosphatidylinositol glycan anchor biosynthesis, class C [Source:MGI Symbol;Acc:MGI:1914542] |
| ENSMUSG00000038729 | 8.200160774 | 0 | 5.440228668 | 0.018710531 | 1 | Pakap | 4 | 8368 | paralemmin A kinase anchor protein [Source:MGI Symbol;Acc:MGI:5141924] |
| ENSMUSG00000062012 | 17.65345851 | 1.694930928 | 3.244978649 | 0.047864424 | 0.45310933 | Zfp13 | 17 | 2652 | zinc finger protein 13 [Source:MGI Symbol;Acc:MGI:99159] |
| ENSMUSG00000068874 | 579.5350076 | 160.183622 | 1.85249875 | 0.001349803 | 0.095570848 | Selenbp1 | 3 | 2273 | selenium binding protein 1 [Source:MGI Symbol;Acc:MGI:96825] |
| ENSMUSG00000032440 | 41.15879973 | 11.05425113 | 1.948567227 | 0.035089684 | 0.390085786 | Tgfbr2 | 9 | 8187 | transforming growth factor, beta receptor II [Source:MGI Symbol;Acc:MGI:98729] |
| ENSMUSG00000003847 | 31.482686 | 6.459838667 | 2.307236792 | 0.018815664 | 0.298485777 | Nfat5 | 8 | 18378 | nuclear factor of activated T cells 5 [Source:MGI Symbol;Acc:MGI:1859333] |
| ENSMUSG00000051034 | 8.473152028 | 0 | 5.485570263 | 0.01713409 | 1 | Zfp11 | 5 | 3929 | zinc finger protein 11 [Source:MGI Symbol;Acc:MGI:99156] |
| ENSMUSG00000035799 | 11.3709388 | 0 | 5.905741723 | 0.014590398 | 0.266185248 | Twist1 | 12 | 1628 | twist basic helix-loop-helix transcription factor 1 [Source:MGI Symbol;Acc:MGI:98872] |
| ENSMUSG00000079465 | 6.713701494 | 0 | 5.160235785 | 0.036359788 | 1 | Col4a3 | 1 | 9967 | collagen, type IV, alpha 3 [Source:MGI Symbol;Acc:MGI:104688] |
| ENSMUSG00000008384 | 21.34066709 | 1.210664948 | 3.964572527 | 0.006637314 | 0.192513957 | Sertad1 | 7 | 1344 | SERTA domain containing 1 [Source:MGI Symbol;Acc:MGI:1913438] |
| ENSMUSG00000000805 | 14.5693486 | 0 | 6.26981872 | 0.001215002 | 0.09047829 | Car4 | 11 | 1966 | carbonic anhydrase 4 [Source:MGI Symbol;Acc:MGI:1096574] |
| ENSMUSG00000018339 | 263.0256924 | 41.34790591 | 2.671148273 | 0.000257703 | 0.043373042 | Gpx3 | 11 | 1813 | glutathione peroxidase 3 [Source:MGI Symbol;Acc:MGI:105102] |
| ENSMUSG00000059475 | 7.52814738 | 0 | 5.335616316 | 0.025705731 | 1 | Zfp426 | 9 | 4888 | zinc finger protein 426 [Source:MGI Symbol;Acc:MGI:1920248] |
| ENSMUSG00000031803 | 8.24622583 | 0 | 5.451117076 | 0.018128955 | 1 | B3gnt3 | 8 | 3055 | UDP-GlcNAc:betaGal beta-1,3-N-acetylglucosaminyltransferase 3 [Source:MGI Symbol;Acc:MGI:2152535] |
| ENSMUSG00000030852 | 61.3033189 | 9.046741563 | 2.715688597 | 0.003405138 | 0.150728323 | Tacc2 | 7 | 15793 | transforming, acidic coiled-coil containing protein 2 [Source:MGI Symbol;Acc:MGI:1928899] |
| ENSMUSG00000015932 | 269.8939497 | 129.792916 | 1.062139358 | 0.023550554 | 0.316601458 | Dstn | 2 | 1911 | destrin [Source:MGI Symbol;Acc:MGI:1929270] |
| ENSMUSG00000029269 | 23.57253042 | 3.797792191 | 2.597332405 | 0.036693895 | 0.396794443 | Sult1b1 | 5 | 2780 | sulfotransferase family 1B, member 1 [Source:MGI Symbol;Acc:MGI:2136282] |
| ENSMUSG00000057315 | 29.93171276 | 2.663462886 | 3.37351703 | 0.009687709 | 0.22203334 | Arhgap24 | 5 | 8792 | Rho GTPase activating protein 24 [Source:MGI Symbol;Acc:MGI:1922647] |
| ENSMUSG00000022623 | 25.06164934 | 2.905595876 | 3.001883385 | 0.031746947 | 0.372188161 | Shank3 | 15 | 9236 | SH3 and multiple ankyrin repeat domains 3 [Source:MGI Symbol;Acc:MGI:1930016] |
| ENSMUSG00000007122 | 15.08019004 | 1.293563622 | 3.50157771 | 0.036004205 | 0.39363053 | Casq1 | 1 | 1951 | calsequestrin 1 [Source:MGI Symbol;Acc:MGI:1309468] |
| ENSMUSG00000052962 | 53.34623171 | 12.38334429 | 2.058174042 | 0.025962341 | 0.333501135 | Mrpl35 | 6 | 4171 | mitochondrial ribosomal protein L35 [Source:MGI Symbol;Acc:MGI:1913473] |
| ENSMUSG00000025491 | 82.9048442 | 21.95351718 | 1.920770195 | 0.007914539 | 0.206908665 | Ifitm1 | 7 | 1094 | interferon induced transmembrane protein 1 [Source:MGI Symbol;Acc:MGI:1915963] |
| ENSMUSG00000034235 | 13.89465995 | 0.24213299 | 5.263993041 | 0.006650691 | 0.192513957 | Usp54 | 14 | 9068 | ubiquitin specific peptidase 54 [Source:MGI Symbol;Acc:MGI:1926037] |
| ENSMUSG00000024049 | 22.10543855 | 1.937063917 | 3.3890795 | 0.018276462 | 0.294977631 | Myom1 | 17 | 10459 | myomesin 1 [Source:MGI Symbol;Acc:MGI:1341430] |
| ENSMUSG00000038569 | 10.35719849 | 0 | 5.789434291 | 0.00808095 | 0.208058217 | Rad9b | 5 | 4322 | RAD9 checkpoint clamp component B [Source:MGI Symbol;Acc:MGI:2385231] |
| ENSMUSG00000045538 | 33.79636779 | 1.937063917 | 3.965954027 | 0.001489607 | 0.10067546 | Ddx28 | 8 | 2262 | DEAD box helicase 28 [Source:MGI Symbol;Acc:MGI:1919236] |
| ENSMUSG00000021223 | 11.15978626 | 0.484265979 | 4.24208662 | 0.028338648 | 0.348761417 | Papln | 12 | 5635 | papilin, proteoglycan-like sulfated glycoprotein [Source:MGI Symbol;Acc:MGI:2386139] |
| ENSMUSG00000032311 | 21.51369716 | 0.726398969 | 4.663942028 | 0.002567098 | 0.127764137 | Nrg4 | 9 | 6075 | neuregulin 4 [Source:MGI Symbol;Acc:MGI:1933833] |
| ENSMUSG00000020593 | 94.74075115 | 16.07516129 | 2.532636031 | 0.004676092 | 0.163706506 | Lpin1 | 12 | 9930 | lipin 1 [Source:MGI Symbol;Acc:MGI:1891340] |
| ENSMUSG00000026960 | 9.601498396 | 0 | 5.669493977 | 0.009782292 | 0.223194079 | Arl6ip6 | 2 | 2703 | ADP-ribosylation factor-like 6 interacting protein 6 [Source:MGI Symbol;Acc:MGI:1929507] |
| ENSMUSG00000006818 | 257.0709971 | 115.5427879 | 1.155773986 | 0.039337711 | 0.408308964 | Sod2 | 17 | 10008 | superoxide dismutase 2, mitochondrial [Source:MGI Symbol;Acc:MGI:98352] |
| ENSMUSG00000050229 | 29.72280219 | 3.474401286 | 3.026439018 | 0.015186795 | 0.271635326 | Pigm | 1 | 7554 | phosphatidylinositol glycan anchor biosynthesis, class M [Source:MGI Symbol;Acc:MGI:1914806] |
| ENSMUSG00000030557 | 57.54130032 | 10.73369305 | 2.392867645 | 0.005291326 | 0.175380286 | Mef2a | 7 | 14681 | myocyte enhancer factor 2A [Source:MGI Symbol;Acc:MGI:99532] |
| ENSMUSG00000004347 | 8.533750449 | 0.323390905 | 4.535800801 | 0.044153816 | 1 | Pde1c | 6 | 20520 | phosphodiesterase 1C [Source:MGI Symbol;Acc:MGI:108413] |
| ENSMUSG00000006273 | 86.93909898 | 28.0328697 | 1.630091358 | 0.022190839 | 0.312378732 | Atp6v1b2 | 8 | 5305 | ATPase, H+ transporting, lysosomal V1 subunit B2 [Source:MGI Symbol;Acc:MGI:109618] |
| ENSMUSG00000018927 | 507.0972647 | 155.1591828 | 1.713796541 | 0.004480409 | 0.161010457 | Ccl6 | 11 | 1795 | chemokine (C-C motif) ligand 6 [Source:MGI Symbol;Acc:MGI:98263] |
| ENSMUSG00000055067 | 9.733313063 | 0 | 5.702414617 | 0.022333948 | 0.312763017 | Smyd3 | 1 | 9184 | SET and MYND domain containing 3 [Source:MGI Symbol;Acc:MGI:1916976] |
| ENSMUSG00000026648 | 10.65988442 | 0 | 5.83970302 | 0.031836683 | 0.372367099 | Dclre1c | 2 | 9316 | DNA cross-link repair 1C [Source:MGI Symbol;Acc:MGI:2441769] |
| ENSMUSG00000012076 | 17.34482535 | 0.24213299 | 5.579060098 | 0.001879454 | 0.111224379 | Brms1l | 12 | 3455 | breast cancer metastasis-suppressor 1-like [Source:MGI Symbol;Acc:MGI:1196337] |
| ENSMUSG00000005505 | 9.53513181 | 0 | 5.660538048 | 0.023308892 | 0.315492781 | Kbtbd4 | 2 | 3139 | kelch repeat and BTB (POZ) domain containing 4 [Source:MGI Symbol;Acc:MGI:1914386] |
| ENSMUSG00000019558 | 14.72301125 | 0.24213299 | 5.340028104 | 0.003822981 | 0.155000706 | Slc6a8 | X | 5210 | solute carrier family 6 (neurotransmitter transporter, creatine), member 8 [Source:MGI Symbol;Acc:MGI:2147834] |
| ENSMUSG00000033161 | 325.1391113 | 116.9482167 | 1.472294694 | 0.005349897 | 0.176352555 | Atp1a1 | 3 | 4568 | ATPase, Na+/K+ transporting, alpha 1 polypeptide [Source:MGI Symbol;Acc:MGI:88105] |
| ENSMUSG00000030905 | 10.82867716 | 0 | 5.862731295 | 0.007003091 | 0.195762618 | Crym | 7 | 3391 | crystallin, mu [Source:MGI Symbol;Acc:MGI:102675] |
| ENSMUSG00000024327 | 15.93038933 | 1.293563622 | 3.580858307 | 0.031527447 | 0.372042643 | Slc39a7 | 17 | 2584 | solute carrier family 39 (zinc transporter), member 7 [Source:MGI Symbol;Acc:MGI:95909] |
| ENSMUSG00000025498 | 640.5576459 | 265.6894416 | 1.271578517 | 0.005042973 | 0.171389035 | Irf7 | 7 | 3776 | interferon regulatory factor 7 [Source:MGI Symbol;Acc:MGI:1859212] |
| ENSMUSG00000020429 | 412.2453676 | 93.23973362 | 2.141548585 | 0.005374973 | 0.176410366 | Igfbp1 | 11 | 1525 | insulin-like growth factor binding protein 1 [Source:MGI Symbol;Acc:MGI:96436] |
| ENSMUSG00000027001 | 17.10027288 | 1.049789874 | 3.875178227 | 0.008928853 | 0.216475808 | Dusp19 | 2 | 2992 | dual specificity phosphatase 19 [Source:MGI Symbol;Acc:MGI:1915332] |
| ENSMUSG00000021068 | 27.64410067 | 3.149369623 | 3.037064668 | 0.010189248 | 0.227603672 | Nin | 12 | 17095 | ninein [Source:MGI Symbol;Acc:MGI:105108] |
| ENSMUSG00000039050 | 31.84451978 | 6.061528529 | 2.367977093 | 0.035133082 | 0.390092662 | Osbpl2 | 2 | 4187 | oxysterol binding protein-like 2 [Source:MGI Symbol;Acc:MGI:2442832] |
| ENSMUSG00000018593 | 274.7047381 | 148.5378789 | 0.889203833 | 0.04241252 | 0.424924462 | Sparc | 11 | 3788 | secreted acidic cysteine rich glycoprotein [Source:MGI Symbol;Acc:MGI:98373] |
| ENSMUSG00000030790 | 6.845822329 | 0 | 5.204039294 | 0.037538407 | 1 | Adm | 7 | 1619 | adrenomedullin [Source:MGI Symbol;Acc:MGI:108058] |
| ENSMUSG00000029924 | 20.15687813 | 2.986853792 | 2.663927746 | 0.047494604 | 0.450876546 | Slc37a3 | 6 | 5409 | solute carrier family 37 (glycerol-3-phosphate transporter), member 3 [Source:MGI Symbol;Acc:MGI:1919394] |
|  |  |  |  |  |  |  |  |  |  |
